# Tailoring of Energy Levels in D-*π*-A Organic Dyes via Fluorination of Acceptor Units for Efficient Dye-Sensitized Solar Cells

**DOI:** 10.1038/srep07711

**Published:** 2015-01-16

**Authors:** Min-Woo Lee, Jae-Yup Kim, Hae Jung Son, Jin Young Kim, BongSoo Kim, Honggon Kim, Doh-Kwon Lee, Kyungkon Kim, Duck-Hyung Lee, Min Jae Ko

**Affiliations:** 1 Photo-Electronic Hybrids Research Center, Korea Institute of Science and Technology, Seoul, 136-791, Korea; 2 Department of Chemistry, Sogang University, Seoul, 121-742, Korea; 3 Department Chemistry and Nano Science, Ewha Womans University, Seoul, 120-750, Korea; 4 Green School, Korea University, 145, Anam-ro, Seongbuk-gu, Seoul 136-701, Korea

## Abstract

A molecular design is presented for tailoring the energy levels in D-*π*-A organic dyes through fluorination of their acceptor units, which is aimed at achieving efficient dye-sensitized solar cells (DSSCs). This is achieved by exploiting the chemical structure of common D-*π*-A organic dyes and incorporating one or two fluorine atoms at the *ortho*-positions of the cyanoacetic acid as additional acceptor units. As the number of incorporated fluorine atoms increases, the LUMO energy level of the organic dye is gradually lowered due to the electron-withdrawing effect of fluorine, which ultimately results in a gradual reduction of the HOMO-LUMO energy gap and an improvement in the spectral response. Systematic investigation of the effects of incorporating fluorine on the photovoltaic properties of DSSCs reveals an upshift in the conduction-band potential of the TiO_2_ electrode during impedance analysis; however, the incorporation of fluorine also results in an increased electron recombination rate, leading to a decrease in the open-circuit voltage (*V*_oc_). Despite this limitation, the conversion efficiency is gradually enhanced as the number of incorporated fluorine atoms is increased, which is attributed to the highly improved spectral response and photocurrent.

Over the last two decades, dye-sensitized solar cells (DSSCs) have attracted considerable attention as a promising candidate for the next generation of solar cells due to their low production cost, high conversion efficiency and ease of processing^1^,^2^,^3^,^4^. A key element of DSSCs is their photosensitizing dye, which fundamentally determines the light-absorption range and light-harvesting efficiency^2^,^5^,^6^,^7^,^8^. The use of ruthenium polypyridine complexes as a photosensitizing dye has allowed conversion efficiencies of more than 11% to be achieved, which results from the broad absorption spectra created by metal-to-ligand charge transfer (MLCT) and favorable photovoltaic properties^5^,^6^,^9^,^10^,^11^. However, their widespread application has been limited by the scarcity and high cost of Ru, problems associated with isomerization or decomposition during the purification process, and a low molar extinction coefficient^12^,^13^. Metal-free organic dyes, on the other hand, offer a number of advantages over Ru-complex dyes, such as a lower material cost, higher molar extinction coefficient, and greater ease of purification^6^. Furthermore, it is typically easier to design and modify the molecular structure of organic dyes formed with a donor-*π* bridge-acceptor (D-π-A) configuration, at least as far as the tuning of the highest occupied molecular orbital (HOMO) and lowest unoccupied molecular orbital (LUMO) energy levels is concerned^13^,^14^,^15^,^16^,^17^,^18^,^19^. Yet in spite of these advantages, the conversion efficiency of organic dyes is still lower than that of Ru-complex dyes due to their narrow absorption spectra and poor photovoltaic properties. Furthermore, they are also plagued by issues of aggregation and a poor stability when used as sensitizers in DSSCs^13^.

To overcome the limitations of organic dyes, and in particular broaden their light-absorption range toward longer wavelengths, various molecular designs have been proposed such as the extension of *π*-conjugated bonding bridges and the incorporation of additional donor or acceptor groups to create a D-D-π-A or D-A-π-A configuration^13^,^14^,^15^,^16^,^17^,^18^,^19^. Mathew et al. also recently demonstrated an efficient Zn-based porphyrin D-π-A dye by introducing a benzothiadiazole group as an additional acceptor, and although this is not a metal-free organic dye, it nonetheless enabled a record DSSC efficiency of 13%^20^. These approaches succeeded in decreasing the HOMO-LUMO energy gap of organic dyes, thereby leading to a broader range of light absorption; however, it has been reported that the extension of π-conjugated bonding bridges causes unfavorable *π*-stacks that result in low stability under illumination^14^,^21^. In addition, most prior research has focused solely on the conversion efficiency of the dye, and so the synthesis methods have tended to be complex and multi-stage processes that are unsuitable for commercial application. There is therefore a need to extend and expand the design options available for the development of more efficient and practical organic dyes.

In light of the limitations of existing organic dyes, we propose a simple molecular design to tailor the energy levels in D-π-A organic dyes through the fluorination of their acceptor units, with the aim of creating more efficient dye-sensitizers for solar cells. Through this, we demonstrate that the LUMO energy level of the D-π-A organic sensitizer can be gradually lowered by increasing the number of fluorine atoms introduced as additional acceptor units due to their electron-withdrawing ability. Using the chemical structure of a common D-π-A organic dye (M5) as a basis, monofluoro- and difluoro-substituted organic dyes are synthesized using a simple method involving commercially available fluorobenzene derivatives, and are designated as M6 and M7, respectively (Fig. 1). The incorporated fluorine is intended to act as an additional acceptor unit to promote electron transfer to the anchoring group by means of its electron-withdrawing effect, which should result in a reduced HOMO-LUMO energy gap^22^. As a result, the synthesized D-π-A-A dyes exhibit a significantly improved spectral response that ensures a higher conversion efficiency than can be achieved with unmodified D-π-A organic dyes. Although there have been several studies in the past pertaining to the fluorination of organic dyes for DSSCs^22^,^23^,^24^,^25^, these all introduced fluorine at a single position within the molecular structure of the organic dye. Thus, to the best of our knowledge, this is the first demonstration that both the spectral response and conversion efficiency can be improved by increasing the incorporation of fluorine (i.e., monofluoro- and difluoro-substitution). We therefore herein discuss the gradual change in the optical and photovoltaic properties of the synthesized organic dyes as the number of incorporated fluorine atoms increases, and explore the effect this has on the energetic and kinetic characteristics of the photoanode in DSSCs.

## Results

**Figure 1:**
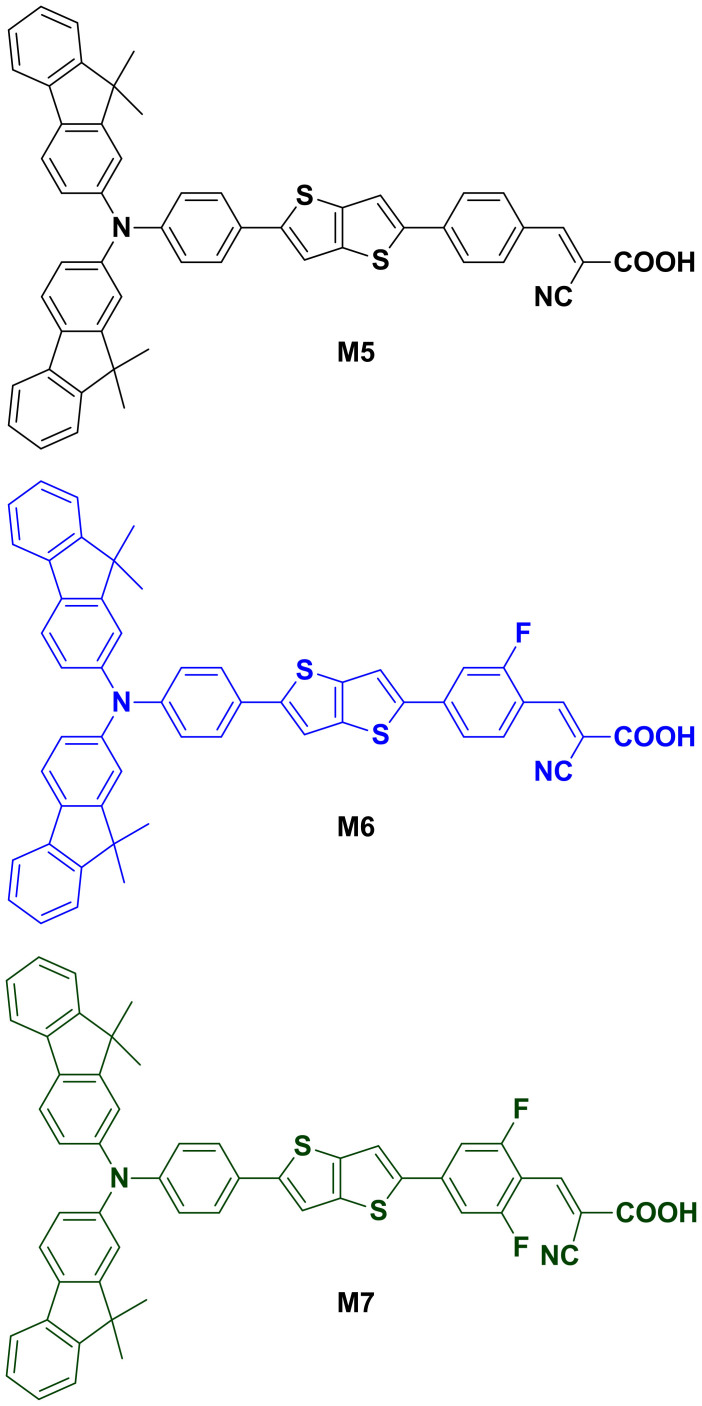
Chemical structure of M series dyes.

The structures and synthesis routes for the M series organic dyes investigated are shown in Fig. 1 and Supplementary Scheme S1, respectively. Note that M5 was designed to have a common D-π-A configuration and therefore serves as a reference dye for M6 and M7, both of which were designed to have a D-π-A-A configuration by incorporating one (for M6) or two (for M7) fluorine atoms at the *ortho*-positions of the cyanoacetic acid as an additional acceptor unit. All dyes were synthesized by a traditional reaction method, full details of which are provided in the Supplementary Information. In brief, starting material **1** (Supplementary Scheme S1) was synthesized from fluorene through multiple steps^26^,^27^, and from this compound **2** was synthesized with thieno[3,2-b]thiophen-2-ylboronic acid using the Suzuki reaction. Compound **3** was synthesized from compound **2** by bromination using *N*-bromosuccinimide (NBS), and was then reacted with 4-formylphenylboronic acid, 3-fluoro-4-formylphenylboronic acid and 3,5-difluoro-4-formylphenylboronic acid via the Suzuki reaction to yield the benzaldehyde derivatives **4**, **5,** and **6**, respectively. The final compounds M5, M6 and M7 were synthesized from the benzaldehyde derivatives **4**, **5**, and **6**, respectively, by the Knoevenagel reaction using cyanoacetic acid with piperidine (for M5 and M6) or ammonium acetate (for M7). The chemical structures of the synthesized organic dyes were characterized and confirmed by ^1^H NMR, ^13^C NMR and mass spectroscopy (shown in the Supplementary Information).

**Figure 2:**
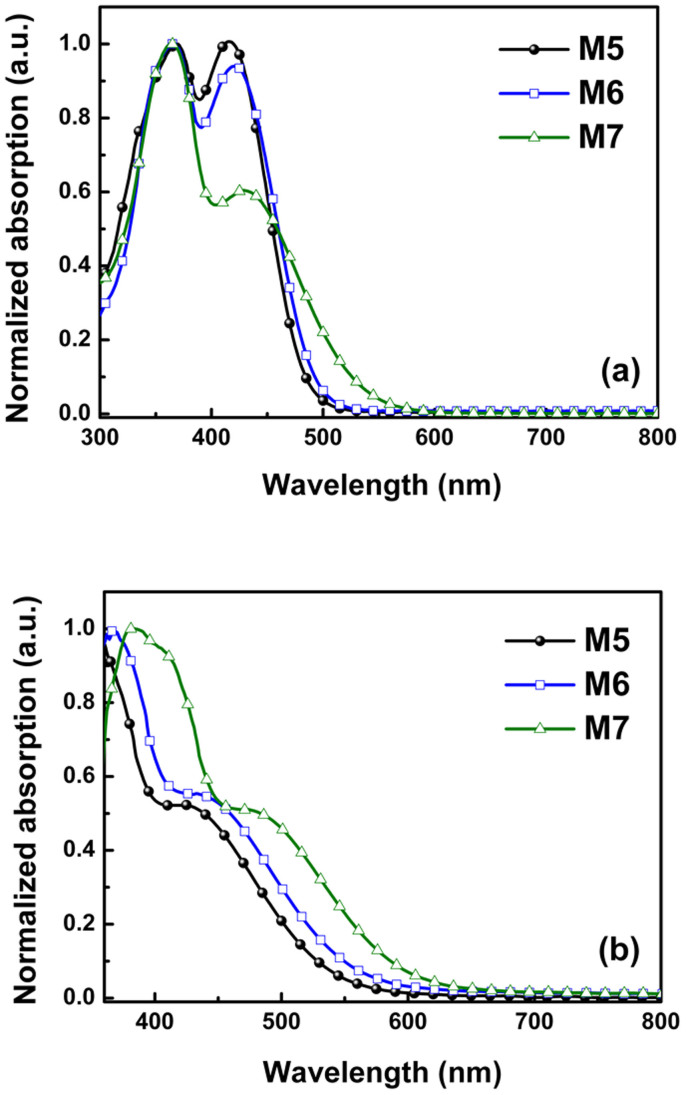
Optical properties of M series dyes. (a) UV-vis absorption spectra in THF. (b) UV-vis absorption spectra on 2-μm-thick TiO_2_ films.

The UV-vis absorption spectra of the M series dyes in THF are shown in Fig. 2a, and their corresponding optical data is listed in Table 1. Generally speaking, D-π-A organic dyes exhibit two major absorption bands, namely the intramolecular charge transfer (ICT) band in the visible region and the local π–π* absorption band in the UV region^28^,^29^. The M series dyes, however, also exhibit two distinct absorption bands in the UV (360–370 nm) and visible region (410–430 nm), with ICT absorption peaks (*λ*_max_) observed at 415, 422 and 428 nm for M5, M6 and M7, respectively. This clearly demonstrates that in the case of organic dyes with a D-π-A-A configuration (i.e., M6 and M7), the absorption peak and onset are red-shifted in comparison to the absorption behavior of the more common D-π-A configuration (M5). Furthermore, even the only difference in chemical structure between M6 and M5 is the presence of one additional electron-withdrawing unit, i.e., fluorine at the *ortho*-position of the cyanoacetic acid (Fig. 1), this is nevertheless sufficient to induce a 7 nm red-shift in the ICT absorption peak. What is more, the two fluorine atoms incorporated at *ortho*-positions in M7 create an even greater red-shift of 13 nm. This gradual increase in the red-shift of the absorption peak with the number of incorporated fluorine atoms can be attributed to a lowering of the LUMO energy level due to the electron-withdrawing effect of fluorine^22^; and as a result, the HOMO-LUMO energy gap (*E*_0-0_) is also reduced from 2.57 eV in M5 to 2.5 and 2.30 eV in M6 and M7, respectively. Moreover, as shown in Table 1, a difluoro-substituted dye (M7) exhibits not only the most extended absorption range, but also has an enhanced molar extinction coefficient (ε) when compared to a monofluoro-substituted dye (M6).

Figure 2b shows the UV-vis absorption spectra of the M series dyes when applied to transparent, 2-*μ*m-thick TiO_2_ films, and are therefore more closely related to the photovoltaic properties than the absorption spectra measured in solution. When adsorbed on a nanocrystalline TiO_2_ surface, deprotonation and *H*-aggregation of the dye molecules typically results in a blue-shift of the absorption spectra, whereas *J*-aggregation results in a red-shift^15^,^30^,^31^. Given that the absorption onset of M5, M6, and M7 on TiO_2_ films was red-shifted by 126, 163, and 180 nm, respectively, when compared to their absorption spectra in THF (Supplementary Fig. S25), *J*-aggregation was clearly dominant^15^,^30^. It is therefore conjectured that this gradual red-shift with increasing number of incorporated fluorine atoms is caused by the fact that fluorine in a benzene moiety enhances the *J*-aggregation between dye molecules. This greater red-shift is advantageous for solar cell applications, as wider range of wavelengths adsorbed should result in a higher photocurrent.

**Figure 3:**
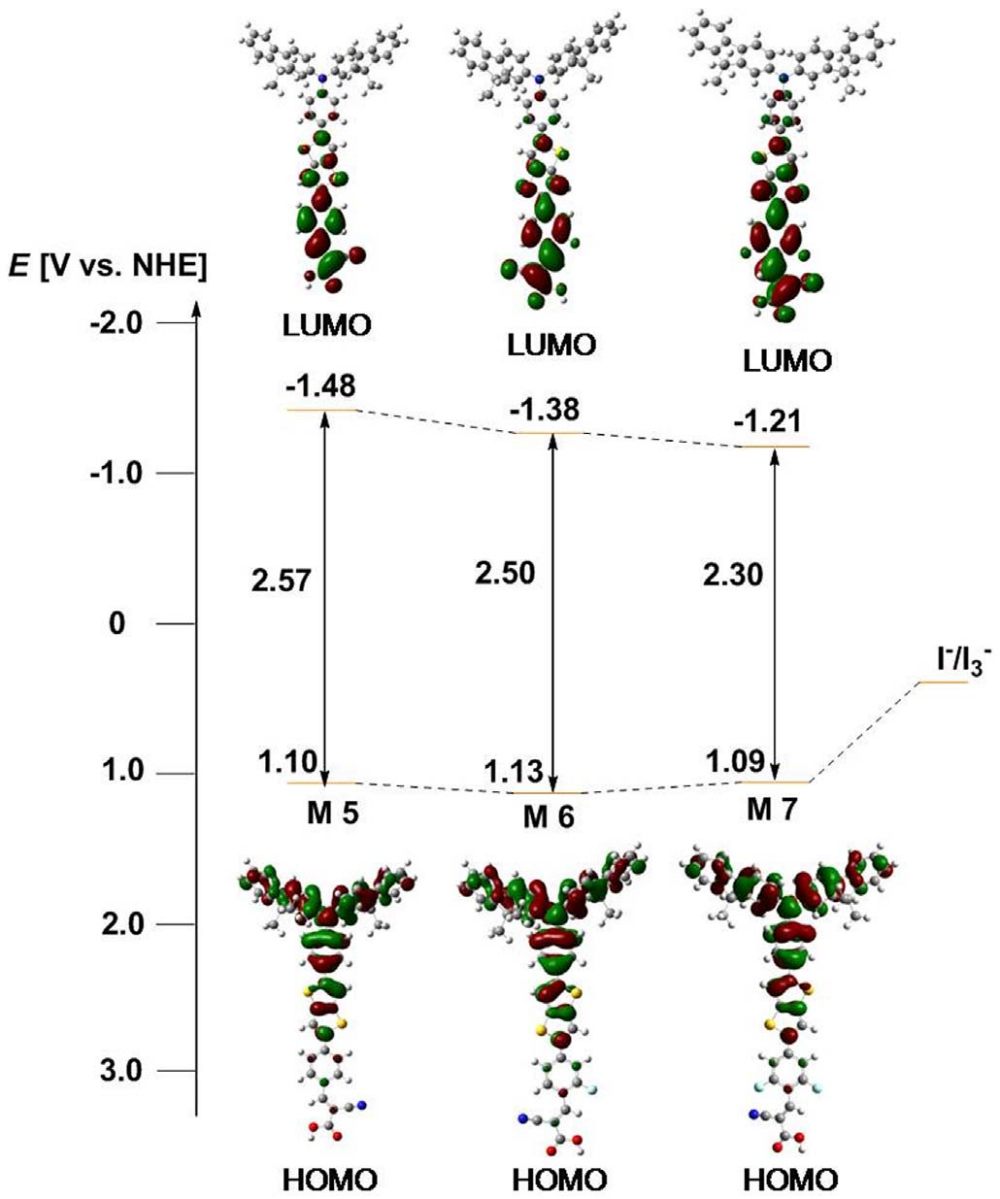
Calculated frontier molecular orbitals and experimental energy-level diagrams of the M series dyes.

To compare the electronic structures of the M series dyes, the HOMO and LUMO electron density surfaces for their frontier molecular orbitals were analyzed by density functional theory (DFT) using the hybrid B3LYP^32^ function with a 6-31G*^33^,^34^ basis set. As shown in Fig. 3, electrons are uniformly delocalized from the donor to the π-bridge at the HOMO level in all of the investigated dyes, which is favorable for an efficient electron transfer^15^,^35^. Meanwhile, at the LUMO level, the excited electron is shifted toward the cyanoacrylic acid acceptor unit and its adjacent benzene moiety. Since the M series has the same molecular structure with respect to the donor part and π-bridge, the electron density distribution at the HOMO level should be similar for each dye. Indeed, the calculated HOMO energy levels (*E*_HOMO_) are very similar at –4.95, –5.00 and –5.03 eV (versus a vacuum) for M5, M6 and M7, respectively. At the LUMO level, on the other hand, the electron density distribution varies slightly due to the additional acceptor unit. The LUMO of M6 has a greater contribution from the cyanoacrylic acid acceptor unit than is seen in M5, while the LUMO of M7 exhibits a further increase in the contribution from this acceptor unit. This trend indicates that the electron-withdrawing effect of fluorine leads to a more effective charge separation between the donor and acceptor part^22^. Consequently, the calculated LUMO energy level (*E*_LUMO_) gradually decreases with an increasing number of incorporated fluorine atoms, giving a value of –2.65, –2.74 and –2.80 eV (versus vacuum) for M5, M6 and M7, respectively. This trend in the calculated *E*_HOMO_ and *E*_LUMO_ values matches well with the experimental data obtained from cyclic voltammograms (CVs) and UV-vis spectra listed in Table 1. For example, the *E*_HOMO_ values determined from the oxidation potential of each dye were all very similar at 1.10, 1.13 and 1.09 V (versus NHE) for M5, M6 and M7, respectively. Similar agreement is also evident in the *E*_LUMO_ values estimated from the 0-0 transition energy (*E*_0-0_) of –1.47, –1.38 and –1.21 V (versus NHE) for M5, M6 and M7, respectively. As predicted by the DFT calculations, the magnitude of *E*_LUMO_ gradually lowers as the electron-withdrawing power of the acceptor part of the dye molecule is enhanced by additional acceptor units. This means that compared to the conduction-band potential (*E*_cb_) of TiO_2_ (–0.28 V versus NHE)^36^, each dye has a sufficiently high *E*_LUMO_ to meet the minimum energy difference (~0.4 eV) needed for effective electron injection from a dye to the conduction band of TiO_2_^37^.

Since the dipole moment of the sensitizer affects the energetics of TiO_2_ electrodes in DSSCs^38^,^39^,^40^, the magnitude of the dipole moment for the M series dyes was evaluated from an optimized geometrical structure (Supplementary Fig. S26) and the resulting values are listed in Supplementary Table S1. Among the three dipole components along each axis, it is the one normal to the surface (*μ*_z_) that can induce a shift in the conduction-band potential of the TiO_2_ electrode^38^,^40^. Moreover, as all of the dyes have a positive dipole moment along their *z*-direction, the negative pole is localized close to the surface of the TiO_2_ electrode when the dye is adsorbed onto it (here, the dipole moment is defined as pointing from the negative to the positive pole). This positive dipole along the *z*-direction can induce an upshift in the conduction-band potential of the TiO_2_ electrode, and this can potentially lead to an increase in the photovoltage of a DSSC^39^,^40^,^41^. As listed in Supplementary Table S1, the magnitude of *μ*_z_ increased in the order of M5 < M6 < M7, with this tendency being attributed to the electron-withdrawing effect of the incorporated fluorine making the acceptor part of the dye molecule more negatively charged.

**Figure 4:**
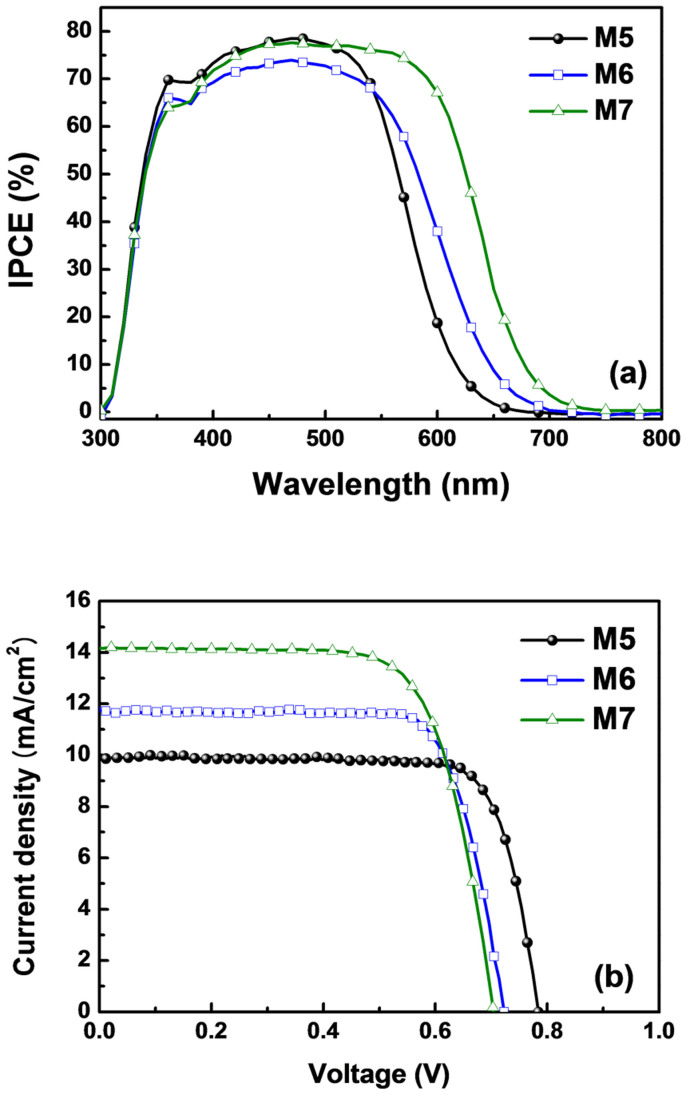
Photovoltaic performance of DSSCs employing an M series dye. (a) IPCE spectra. (b) Photocurrent–voltage (*J*–*V*) characteristics (light intensity: 100 mW/cm^2^, AM 1.5 G filter).

Figure 4a shows the incident photon-to-current conversion efficiency (IPCE) for DSSCs employing the M series dyes as a function of the incident wavelength, which reveals that all three dyes exhibit a high maximum IPCE of 70–80%. The IPCE spectra also exhibit a similar trend to the UV-vis spectra, in that the IPCE spectrum is gradually extended as the number of incorporated fluorine atoms is increased due to the accompanying decrease in *E*_0-0_. The onset wavelength became longer in the order of M5 < M6 < M7; i.e., onset was at about 660, 710, and 750 nm for M5, M6 and M7, respectively. This data provides clear evidence that the reduction in the HOMO-LUMO energy gap that is created by the incorporation of fluorine as an additional acceptor unit can effectively improve the spectral response and photocurrent of DSSCs. This trend also influenced the photocurrent–voltage (*J*–*V*) characteristics, as shown in Fig. 4b and listed in Table 2, with the short-circuit photocurrent (*J*_sc_) being gradually increased from 9.99 mA/cm^2^ with M5 to 11.78 and 14.20 mA/cm^2^ with M6 and M7, respectively. This increase in *J*_sc_ is mainly attributed to the extended wavelength range of light absorption that was confirmed by the IPCE spectra. In contrast, the open-circuit voltage (*V*_oc_) decreased as the number of incorporated fluorine atoms was increased; and since the magnitude of the increase in *J*_sc_ was greater than the decrease in *V*_oc_, the conversion efficiency was subsequently enhanced in the order of M5 < M6 < M7 (6.13, 6.46 and 7.12% for M5, M6, and M7, respectively).

**Figure 5:**
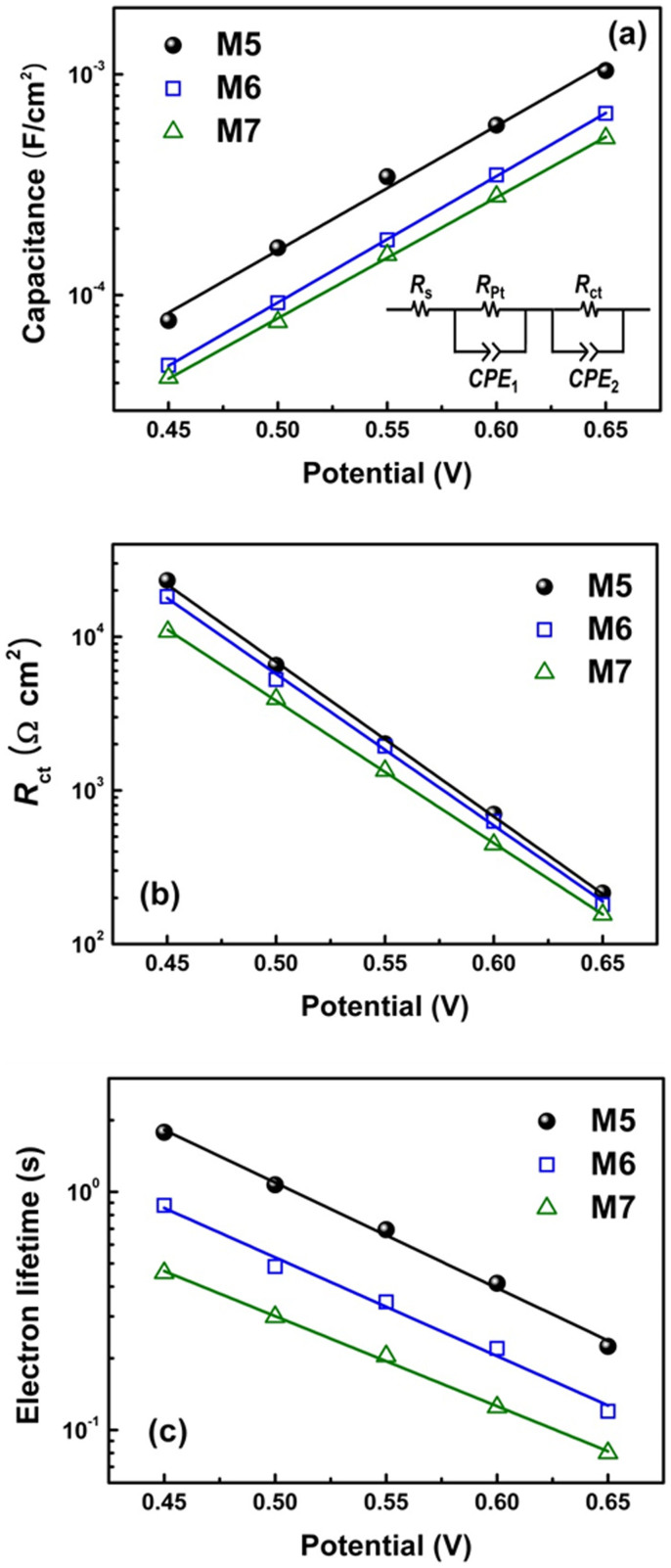
Electrochemical impedance data of DSSCs employing an M series dye. (a) Charge transfer resistance (inset: equivalent circuit model), (b) chemical capacitance, and (c) electron lifetime (as calculated from the dark-state impedance spectra).

The *V*_oc_ exhibited a different trend to what was anticipated from the calculated dipole moment of the M series, with the DFT calculations suggesting that *V*_oc_ would increase in the order of M5 < M6 < M7, whereas the experimental results were in fact the compete opposite. In order to more clearly understand this deviation, the energetic and kinetic characteristics of photoanodes employing M series dyes were examined through electrochemical impedance analysis in a dark state at bias potentials ranging from –0.45 to –0.65 V. As shown in the inset of Fig. 5a, an equivalent-circuit model consisting of a series resistance (*R*_s_) and the impedance at the electrolyte/Pt counter electrode (*R*_Pt_ and CPE_1_) and electrolyte/TiO_2_ electrode (*R*_ct_ and CPE_2_) was used to fit the impedance spectra^42^. Note that CPE indicates a “constant phase element”, and so represents the interfacial capacitance of electrodes with roughness^41^,^43^. Thus, the chemical capacitance (*C*_μ_) of the TiO_2_ film can be evaluated from CPE_2_^41^. The fitting parameters obtained using ZView software are plotted in Fig. 5 as a function of the bias potential. As shown in Fig. 5a, *C*_μ_ increases in the order of M5 < M6 < M7 at the same bias potential; or in other words, for a given chemical capacitance, the bias potential is highest with M7 (i.e., the conduction-band potential of the TiO_2_ electrode is most negative^41^), but lowest with M5. This trend is in good agreement with the calculated results for the dipole moments normal to the TiO_2_ surface (*μ*_z_), which increase in the order of M5 < M6 < M7. In short, as the number of incorporated fluorine atoms in the dye molecule is increased, the magnitude of the dipole moment is also increased and creates further upshift in the conduction-band potential of the TiO_2_ electrode.

As shown in Fig. 5b, however, the interfacial charge transfer resistance (*R*_ct_) for a given bias potential decreases in the order of M5 > M6 > M7, implying that the electron recombination rate becomes faster as the number of incorporated fluorine atoms is increased. Consequently, the electron lifetime (*τ*_n_) also decreases in the order of M5 > M6 > M7, as this is determined by the product of *C*_μ_ and *R*_ct_. To provide a proper comparison of *R*_ct_ and *τ*_n_ without the effects of varying conduction-band potential, plots of *R*_ct_ and *τ*_n_ with a potential displacement for M6 and M7 based on the difference in *C*_μ_ with respect to M5^44^ are presented in Supplementary Fig. S27. This shows that the trend among the M series dyes does not change, but the discrepancies do become larger when compared to the original plots. Nevertheless, it is clear that increasing the number of incorporated fluorine atoms makes electron recombination faster and therefore reduces the electron lifetime was reduced, which may explain the decrease in *V*_oc_. Put simply, incorporating fluorine into the dye framework makes the energetic characteristics (the conduction-band potential) of the photoanode more favorable in terms of a high *V*_oc_, but the dominance of the unfavorable kinetic characteristics (the electron recombination rate) ultimately results in a decrease in *V*_oc_. The precise reason for this increase in electron recombination rate with fluorine incorporation remains unknown at this point, but is likely to be related to a change in the local concentration of I_3_^−^ near the TiO_2_ film^45^ due to the dipole moment of the organic dyes. If so, then one possible way to overcome this limitation would be to introduce suitably bulky alkyl groups into the dye framework, which could reduce electron recombination with the electrolyte due to the blocking effect^46^. Further work is currently being undertaken to develop organic dyes with just such a structure.

## Discussion

New monofluoro- and difluoro-substituted organic dyes were successfully prepared with a D-π-A-A configuration for application in DSSCs. This was made possible by incorporating one or two fluorine atoms at the *ortho*-positions of the cyanoacetic acid in the organic dye to act as an additional acceptor unit and reduce the HOMO-LUMO energy gap. This represents a simple process that requires only commercially available fluorobenzene derivatives. Subsequent CV analyses confirmed that the LUMO energy level is gradually reduced as the electron-withdrawing power of the acceptor part of the dye molecule is enhanced by the incorporation of fluorine. The resulting extension of the light absorption wavelength range leads to a broader IPCE spectrum and an enhanced photocurrent in DSSCs employing D-π-A-A organic dyes when compared to those employing more conventional D-π-A organic dyes. Impedance analyses also revealed that the incorporation of fluorine creates an upshift in the conduction-band potential of the TiO_2_ electrode, but this is accompanied by an increase in the electron recombination rate that ultimately leads to a decrease in *V*_oc_. Yet despite this limitation, the conversion efficiency of DSSCs is gradually enhanced as the number of incorporated fluorine atoms is increased, due largely to a greatly improved photocurrent. These photovoltaic results indicate that the incorporation of fluorine into organic dyes as an additional acceptor unit is a promising design strategy for enhancing the spectral response and conversion efficiency of DSSCs. However, further modification of the molecular design is needed to reduce the electron recombination rate in order to achieve even more efficient photovoltaic properties in fluoro-substituted D-π-A-A organic dyes.

## Methods

### Characterization of organic dyes

CV measurements of the synthesized dyes were conducted using an electrochemical analyzer (CH Instruments Inc., Austin, TX). For this, a saturated calomel electrode (SCE) was used as the reference electrode, and a platinum disk and wire were used as the working and counter electrode, respectively. The solution used for all measurements consisted of 1 mM of dye or ferrocene (Fc) in THF with 0.1 M of tetramethylammonium tetrafluoroborate (TMATFB) as a supporting electrolyte. The redox potentials of the dyes were determined versus Fc at a scan rate of 0.1 Vs^−1^, and were then converted to values relative to a normal hydrogen electrode (NHE) by adding a constant of 0.67 V. The absorption spectra of the dyes dissolved in THF were obtained by UV-vis spectroscopy (Agilent 8453).

### Fabrication of DSSCs

TiO_2_ nanoparticles with a diameter of about 20 nm were synthesized, from which a screen-printable paste was prepared in accordance with a previously reported procedure^47^. Fluorine-doped tin-oxide (FTO) glass pieces (Pilkington, TEC-8) were prepared by washing for 10 min in ethanol using an ultrasonic bath, which was followed by 20 min of UV–O_3_ treatment to remove any residual organic impurities. After pre-treating with 7.5 wt% Ti(IV) bis(ethyl acetoacetato)-diisopropoxide (Aldrich) solution, the TiO_2_ paste was spin coated onto the FTO substrates, then annealed at 500°C for 30 min. A scattering paste containing 500-nm-sized TiO_2_ particles (G2, Showa Denko, Japan) was then deposited on the annealed TiO_2_ films, which was followed by a second annealing using the same heating profile. Next, the electrode was dipped in 0.04 M TiCl_4_ solution at 70°C for 30 min, washed with water, and then annealed at 500°C for 30 min. The thicknesses of the annealed nanocrystalline layer and scattering layer were approximately 7 and 5 μm, respectively, as measured by an Alpha-Step IQ surface profiler (KLA Tencor). After cooling to 80°C, the TiO_2_ electrode was immersed overnight in a chloroform solution containing 0.5 mM of dye. A counter electrode was prepared by spreading a 7 mM H_2_PtCl_6_ solution in 2-propanol onto the FTO glass, then annealing at 400°C for 20 min in air. This Pt counter electrode was then assembled with the dye-adsorbed TiO_2_ electrode using 25-μm-thick Surlyn (Dupont 1702). The electrolyte solution used consisted of 0.6 M tetrabutyl ammonium iodide (TBAI), 0.1 M LiI, 0.05 M I_2_, and 0.5 M 4-*tert*-butylpyridine (TBP) dissolved in a mixed solution of acetonitrile (AN) and valeronitrile (VN) (85/15, v/v). The active area of the dye-coated TiO_2_ film was about 0.450 cm^2^, which was measured by an image analysis program equipped with a CCD camera (moticam 1000).

### Photoelectrochemical measurements

Photocurrent-voltage (*J-V*) measurements were performed using a Keithley model 2400 source measurement unit. A solar simulator (Yamasita) equipped with a 1000 W Xenon lamp was used as the light source, the intensity of which was adjusted using an NREL-calibrated Si solar cell with a KG-5 filter to approximate 1 sun light intensity. A black aperture mask was used to cover the cells during measurement to prevent any additional illumination through the lateral space^48^. The distance between the edges of the photoactive site and mask aperture was 1 mm, with the area of the aperture mask being determined as be 0.78 cm^2^ in a previous study^48^. Incident photon-to-current conversion efficiency (IPCE) data was obtained as a function of wavelength from 300 to 800 nm using an IPCE system specially designed for DSSCs (PV measurements, Inc.), in which a 75 W Xenon lamp is used to generate a monochromatic beam. Calibration was performed using a Si photodiode, which in itself was calibrated by a NIST-calibrated standard photodiode G425. All electrochemical impedance measurements were performed in a dark state at bias potentials ranging from −0.45 to −0.65 V using a Solartron 1287 potentiostat and a Solartron 1260 frequency-response detector. For this, sinusoidal perturbations of 10 mV in magnitude were applied within a frequency range from 10^−1^ to 10^5^ Hz.

## Author Contributions

M.-W.L., J.-Y.K. and M.J.K. designed the research, analyzed the experimental data and co-wrote the paper with the contributions from all authors. M.-W.L. performed the experiments. H.J.S., J.Y.K., B.K., H.K., D.-K.L., K.K. and D.-H.L. contributed to discussion of the results. M.J.K. conceived the research and provided overall guidance.

## Supplementary Material

Supplementary Information Supplementary Information

## Figures and Tables

**Table 1:** Optical and electrochemical data for M series dyes

Dye	*λ*_max_ [nm] a	*ε* [M^−1^cm^−1^] a	*E*_HOMO_ [V] b	*E*_0-0_ [V] c	*E*_LUMO_ [V] d
M5	369	79,500	1.10	2.57	−1.47
	415	80,000			
M6	366	79,204	1.13	2.50	−1.38
	422	74,545			
M7	364	157,540	1.09	2.30	−1.21
	428	95,185			

^a^Recorded in THF solution at 298 K.

^b^Recorded in THF solution at 298 K, potentials measured versus Fc^+^/Fc (Eox = 0.85 V versus Ag/Ag^+^) were converted to values against a normal hydrogen electrode (NHE) by the addition of a constant of +0.63 V and taken as *E*_HOMO_.

^c^0-0 transition energy, *E*_0-0_, determined by using the absorption onset of the UV-vis spectra.

^d^*E*_LUMO_ was calculated by *E*_HOMO_ – *E*_0-0_.

**Table 2:** Summary of the *J–V* characteristics for DSSCs employing M series dyes

Dye	*J*_sc_ (mA/cm^2^)	*V*_oc_ (mV)	Fill factor (%)	Efficiency (%)
M5	9.99	784	78.20	6.12
M6	11.78	723	75.84	6.46
M7	14.20	704	71.41	7.14
