# Supplementary material for: Tailoring of Energy Levels in D-π-A Organic Dyes via Fluorination of Acceptor Units for Efficient Dye-Sensitized Solar Cells
Source: Sci Rep. 2015 Jan 16;5:7711. doi: 10.1038/srep07711 (PMC4296309; doi:10.1038/srep07711)
Supplement: Supplementary Information [file srep07711-s1.pdf]

Supplementary Information

Tailoring of Energy Levels in D-*π*-A Organic Dyes via Fluorination of Acceptor Units for Efficient Dye-Sensitized Solar Cells

Min-Woo Lee[1,2†], Jae-Yup Kim[1†], Hae Jung Son[1], Jin Young Kim[1,4], BongSoo Kim[1,4], Honggon Kim[1,4], Doh-Kwon Lee[1], Kyungkon Kim[3], Duck-Hyung Lee[2], Min Jae Ko[1,4]*

[1]Photo-Electronic Hybrids Research Center, Korea Institute of Science and Technology, Seoul, 136-791, Korea, [2]Department of Chemistry, Sogang University, Seoul, 121-742, Korea, [3]Department Chemistry and Nano Science, Ewha Womans University, Seoul, 120-750, Korea, [4]Green School, Korea University, 145, Anam-ro, Seongbuk-gu, Seoul 136-701, Korea.

*Correspondence and requests for materials should be addressed to M. J. K. (mjko@kist.re.kr)

[†] These authors contributed equally to this work.

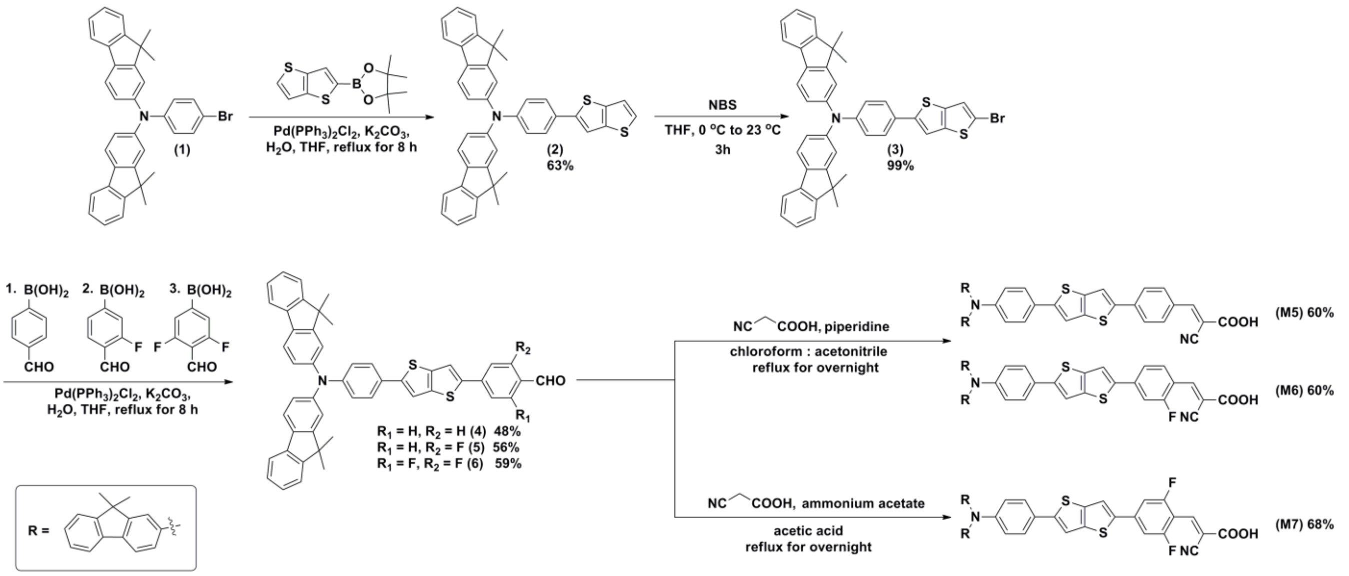

Scheme S1 | Synthesis routes of the M series.

Synthesis of *N*-(9,9-dimethyl-9H-fluoren-2-yl)-9,9-dimethyl-*N*-(4-(thieno[3,2-b]thiophen-2-yl)phenyl)-9H-fluoren-2-amine (2). A mixture of 1 (3.06 g, 5.50 mmol), thieno[3,2-b]thiophen-2-ylboronic acid (1.24 g, 5.50 mmol) and $Pd(PPh_3)_2Cl_2$ (386 mg, 0.55 mmol) in K_2CO_3 (3.3 ml), H_2O (6.72 ml) and dimethoxyethane (18 ml) was stirred at 100 °C overnight. After cooling to room temperature, the reaction mixture was filtered through Celite, and the filtrate was poured into water and extracted with EtOAc. The combined organic phases were washed with brine, dried with Na_2SO_4, and concentrated in vacuo. The crude residue was purified by column chromatography to give adduct (2.14 g, 63%). ^{1}H NMR (300 MHz, CDCl3, δ): 7.67-7.60 (m, 4H), 7.54-7.51 (m, 2H), 7.42-7.39 (m, 4H), 7.26 (brs, 4H), 7.22-7.18 (d, 2H), 7.14 (brs, 2H), 1.42 (s, 12H). ^{13}C NMR (300 MHz, CDCl3, δ): 156.1, 154.4, 148.1, 147.9, 147.9, 139.8, 139.8, 135.5, 135.4, 134.8, 134.7, 134.6, 134.5, 133.2, 133.1, 133.0, 129.6, 129.5, 129.4, 128.0, 127.6, 127.5, 126.3, 126.2, 124.1, 123.8, 123.5, 121.7, 121.6, 120.5, 120.4, 119.6, 115.8, 115.7, 78.2, 78.0, 77.8, 47.8, 47.7, 28.0, 27.9, 27.8 LRMS (ESI, m/z): [M]+ calcd for $C_{42}H_{33}NS_2$, 615; found, 615.38. LRMS (ESI, m/z): [M]+ calcd for $C_{42}H_{33}NS_2$, 615; found, 615.21.

Synthesis of *N*-(4-(5-bromothieno[3,2-b]thiophen-2-yl)phenyl)-*N*-(9,9-dimethyl-9H-fluoren-2-yl)-9,9-dimethyl-9H-fluoren-2-amine (3). NBS (629 mg, 3.53 mmol) was added in one portion to a solution of 2 (1.45 g, 2.35 mmol) in THF (30 mL) at 0 °C. The reaction mixture was stirred at 0 °C for 3 h. The reaction was quenched by the addition of water and extracted with DCM. The combined organic extract was dried over anhydrous $MgSO_4$ and concentrated in vacuo. The crude residue was purified by column chromatography to give adduct

(1.62 g, 99%). ^{1}H NMR (300 MHz, CDCl3, δ): 7.68-7.57 (m, 6H), 7.41-7.39 (d, 3H), 7.33-7.29 (d, 5H), 7.26 (brs, 5H), 7.21-7.15 (m, 3H), 1.42 (s, 12H). ^{13}C NMR (300 MHz, CDCl3, δ): 158.01, 156.6, 156.4, 156.3, 155.7, 155.6, 154.7, 147.5, 146.9, 144.3, 140.1, 138.8, 132.9, 132.8, 130.9, 128.2, 127.9, 127.5, 126.9, 126.8, 126.1, 124.2, 123.9, 123.6, 123.4, 121.8, 121.5, 121.1, 120.4, 118.4, 117.2, 114.3, 78.1, 77.9, 77.7, 47.9, 47.8, 47.7, 30.4, 27.9, 27.8, 27.7 LRMS (ESI, m/z): [M]+ calcd for $C_{42}H_{32}BrNS_2$, 693.12; found, 694.98.

Synthesis of 4-(5-(4-(bis(9,9-dimethyl-9H-fluoren-2-yl)amino)phenyl)thieno[3,2-b]thiophen-2-yl)benzaldehyde (4). Compound 3 (700 mg, 1.01 mmol) was reacted with 4-formylphenylboronic acid (181.4 mg, 1.21 mmol) under Suzuki coupling reaction using $Pd(PPh_3)_2Cl_2$ (70.8 mg, 0.101 mmol) as a catalyst in K_2CO_3 (1.39 g), H_2O (18.8 ml) and THF (78 mL) to yield compound 4 (350 mg, 48%). ^{1}H NMR (300 MHz, CDCl3, δ): 10.02 (s, 1H), 7.91 (s, 2H), 7.80-7.79 (d, 2H), 7.66-7.63 (m, 6H), 7.55-7.52 (d, 1H), 7.43-7.40 (d, 3H), 7.33-7.22 (m, 7H), 7.19-7.12 (m, 3H), 1.43 (s, 12H). ^{13}C NMR (600 MHz, CDCl3, δ): 191.11, 155.22, 153.56, 146.85, 146.70, 140.07, 138.82, 134.90, 134.64, 130.60, 130.52, 129.67, 129.41, 127.22, 126.84, 126.47, 125.84, 125.63, 124.01, 123.66, 122.71, 122.55, 122.33, 120.85, 120.61, 119.96, 119.64, 119.50, 119.42, 119.14, 118.79, 118.02, 117.78, 98.91, 46.87, 27.61, 27.23, 26.84, 26.47. LRMS (ESI, m/z): [M + H]+ calcd for $C_{49}H_{37}NOS_2$, 719.23; found, 720.34.

Synthesis of 4-(5-(4-(bis(9,9-dimethyl-9H-fluoren-2-yl)amino)phenyl)thieno[3,2-b]thiophen-2-yl)-2-fluorobenzaldehyde (5). Compound 3 (700 mg, 1.01 mmol) was reacted with 3-fluoro-4-formylphenylboronic acid (203.2 mg, 1.21 mmol) under Suzuki coupling reaction using $Pd(PPh_3)_2Cl_2$ (70.8 mg, 0.101 mmol) as a catalyst in K_2CO_3 (1.39 g), H_2O (18.8 ml) and THF (78 mL) to yield compound 5 (380 mg, 56%). ^{1}H NMR (300 MHz, CDCl3, δ): 10.35 (s, 1H), 7.91-7.89 (d, 1H), 7.67-7.63 (m, 5H), 7.55-7.53 (d, 2H), 7.40 (s, 4H), 7.34-7.22 (m, 8H), 7.19-7.16 (d, 3H), 1.43 (s, 12H). ^{13}C NMR (600 MHz, CDCl3, δ): 185.30, 165.87, 164.16, 155.23, 153.57, 148.57, 146.80, 146.65, 143.39, 142.36, 142.30, 142.11, 141.33, 138.80, 136.04, 134.94, 129.67, 129.42, 127.23, 126.85, 126.52, 124.05, 123.70, 122.71, 122.48, 122.35, 122.12, 120.87, 120.63, 119.65, 119.53, 119.44, 119.18, 98.85, 46.89, 27.61, 27.23, 26.85, 26.47. LRMS (ESI, m/z): [M + H]+ calcd for $C_{49}H_{36}FNOS_2$, 737.21; found, 738.05.

Synthesis of *N*-(9,9-dimethyl-9H-fluoren-2-yl)-9,9-dimethyl-*N*-(4-(4,4,5,5-tetramethyl-1,3,2-dioxaborolan-

2-yl)phenyl)-9H-fluoren-2-amine (6). Compound 3 (700 mg, 1.01 mmol) was reacted with 3,5-difluoro-4-formylphenylboronic acid (225 mg, 1.21 mmol) under Suzuki coupling reaction using $Pd(PPh_3)_2Cl_2$ (70.8 mg, 0.101 mmol) as a catalyst in K_2CO_3 (1.39 g), H_2O (18.8 ml) and THF (78 mL) to yield compound 6 (412 mg, 59%). ^{1}H NMR (300 MHz, DMSO, δ): 10.16 (s, 1H), 8.31 (s, 1H), 7.86 (s, 1H), 7.80-7.74 (m, 4H), 7.71-7.58 (m, 4H), 7.52-7.50 (d, 2H), 7.35-7.26 (m, 6H), 7.15-7.10 (m, 4H), 1.37 (s, 12H). ^{13}C NMR (600 MHz, DMSO, δ): 184.4, 164.0, 162.3, 155.4, 153.7, 148.6, 146.6, 146.4, 143.3, 141.7, 140.9, 138.6, 138.5, 136.3, 135.0, 134.7, 129.8, 127.5, 127.3, 127.2, 127.0, 125.3, 124.2, 123.8, 123.3, 123.1, 122.0, 121.8, 121.7, 120.1, 120.0, 119.7, 119.2, 109.4, 109.2, 98.8, 79.5, 46.9, 27.0 LRMS (ESI, m/z): [M + H]+ calcd for $C_{49}H_{35}F_2NOS_2$, 755.21; found, 755.38.

Synthesis of M5. A mixture of aldehyde **4** (Scheme S1, Supporting Information, 275.5 mg, 0.383 mmol) and cyanoacetic acid (72.8 mg, 0.856 mmol) were added into the mixed solution of acetonitrile (2.4 ml), chloroform (4.7 ml) and piperidine (10.9 mg, 0.128 mmol) at room temperature, followed by refluxing overnight. After cooling to room temperature, the organic phase was separated and the aqueous layer was extracted by dichloromethane (CH_2Cl_2). The combined organic phases were washed with brine, dried with $MgSO_4$, and concentrated *in vacuo*. The crude residue was purified by column chromatography to give adduct (180 mg, 60%) in form of an orange solid. ^{1}H NMR (300 MHz, DMSO, δ): 8.18 (s, 1H), 8.07-8.00 (d, 4H), 7.87-7.82 (m, 6H), 7.72-7.70 (d, 2H), 7.57-7.55 (d, 2H), 7.40-7.36 (d, 6H), 7.18 (brs, 4H), 1.42 (s, 12H). ^{13}C NMR (600 MHz, DMSO, *δ*): 163.42, 154.91, 154.83, 153.21, 153.16, 147.91, 146.63, 146.31, 146.07, 143.62, 139.52, 138.18, 138.11, 136.11, 135.43, 134.51, 132.86, 130.26, 129.35, 127.05, 126.80, 126.71, 126.42, 125.53, 125.38, 125.30, 123.68, 123.20, 123.13, 122.64, 121.86, 121.23, 121.14, 119.64, 119.57, 119.12, 118.58, 118.51, 98.43, 46.43, 26.63. LRMS (ESI, *m/z*): $[M + H]^+$ calcd for $C_{52}H_{38}N_2O_2S_2$, 786.24; found, 787.00.

Synthesis of M6. M6 was obtained from aldehyde **5** (Scheme S1, Supporting Information) in similar way with **M5** (yield 60%). ^{1}H NMR (300 MHz, DMSO, δ): 8.18 (s, 2H), 8.09 (s, 1H), 7.81-7.76 (m, 3H), 7.74-7.64 (m, 5H), 7.57-7.54 (d, 2H), 7.38-7.32 (m, 6H), 7.20-7.08 (m, 5H), 1.41 (s, 12H). ^{13}C NMR (600 MHz, DMSO, *δ*): 162.38, 161.32, 159.65, 154.87, 154.82, 153.19, 147.90, 146.27, 146.03, 142.12, 141.59, 139.95, 138.17, 138.10, 137.62, 135.88, 134.48, 134.48, 134.12, 129.32, 128.85, 128.89, 127.04, 126.80, 126.71, 126.45, 125.33, 123.64, 123.20, 123.05, 122.64, 121.85, 121.39, 121.19, 121.13, 119.61, 119.28, 119.09, 118.60, 112.03, 98.35, 46.58, 26.61.

LRMS (ESI, *m*/*z*): $[M + H]^+$ calcd for $C_{52}H_{37}FN_2O_2S_2$, 804.23; found, 805.25.

Synthesis of M7. A mixture of aldehyde **6** (Scheme S1, Supporting Information, 250.5 mg, 0.331 mmol) with cyanoacetic acid (72.8 mg, 0.856 mmol) and ammonium acetate (12.8 mg, 0.166 mmol) were added into glacial acetic acid (9 ml) at room temperature, followed by refluxing overnight. After cooling to room temperature, the organic phase was separated and the aqueous layer was extracted by CH_2Cl_2. The combined organic phases were washed with brine, dried with $MgSO_4$, and concentrated in vacuo. The crude residue was purified by column chromatography to give adduct (185 mg, 68%) in form of an orange solid. ^1H NMR (300 MHz, DMSO, δ): 8.33 (s, 1H), 7.77-7.72 (m, 5H), 7.66-7.60 (m, 5H), 7.51-7.49 (d, 2H), 7.34-7.26 (m, 6H), 7.15-7.08 (m, 4H), 1.36 (s, 12H). ^{13}C NMR (300 MHz, DMSO, *δ*): 162.0, 161.0, 159.4, 155.3, 155.3, 153.6, 153.6, 148.5, 146.7, 146.4, 142.3, 141.6, 140.8, 138.6, 138.5, 136.2, 135.0, 134.6, 129.8, 127.5, 127.2, 126.9, 125.6, 124.2, 123.7, 123.4, 123.1, 122.1, 121.7, 121.6, 120.5, 120.1, 120.0, 119.6, 119.2, 119.1, 117.8, 109.1, 108.9, 98.8, 79.6, 46.9, 27.0. LRMS (ESI, *m*/*z*): $[M + H]^+$ calcd for $C_{52}H_{36}F_2N_2O_2S_2$, 822.22; found, 823.33.

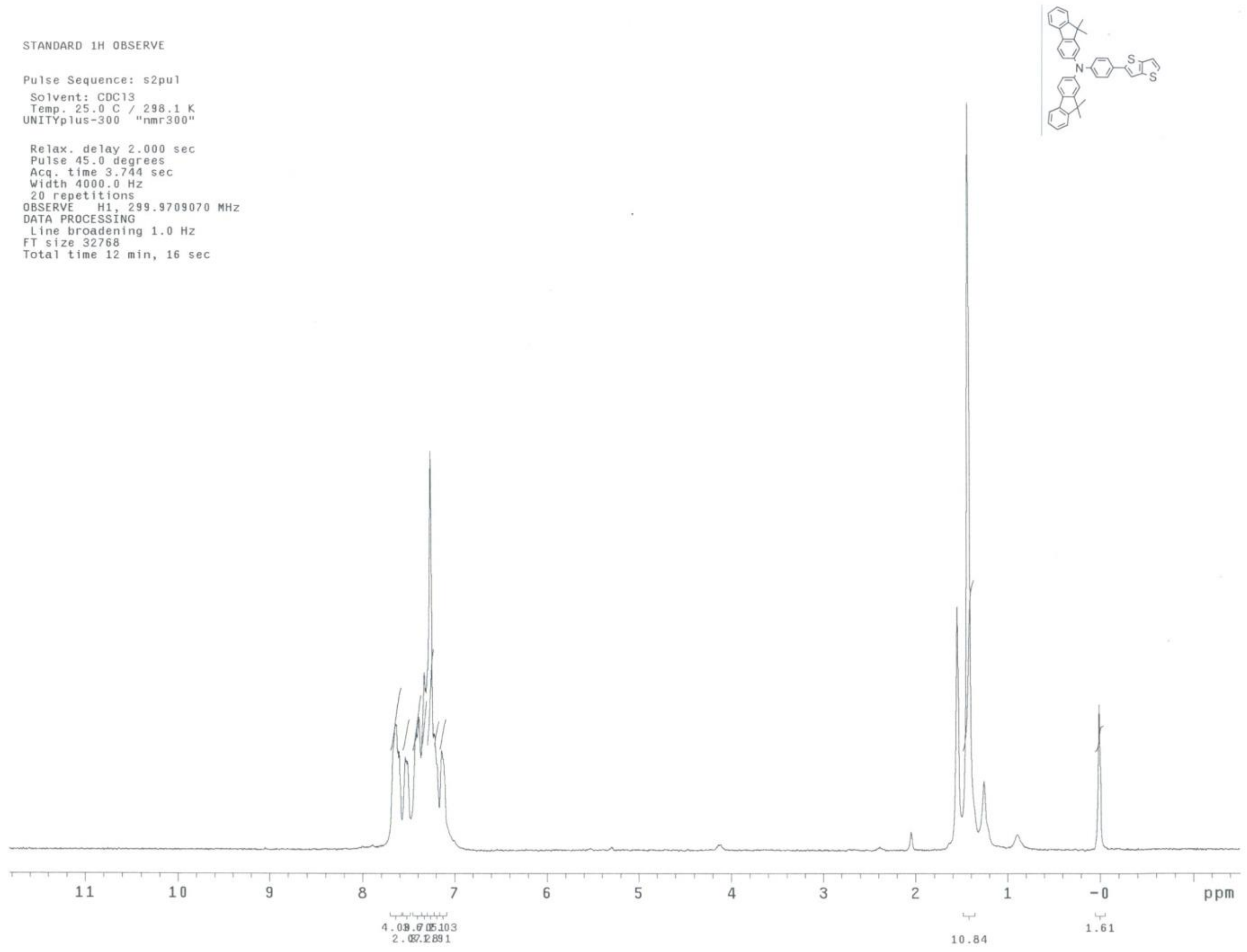

Figure S1 | ^{1}H NMR spectrum of (2).

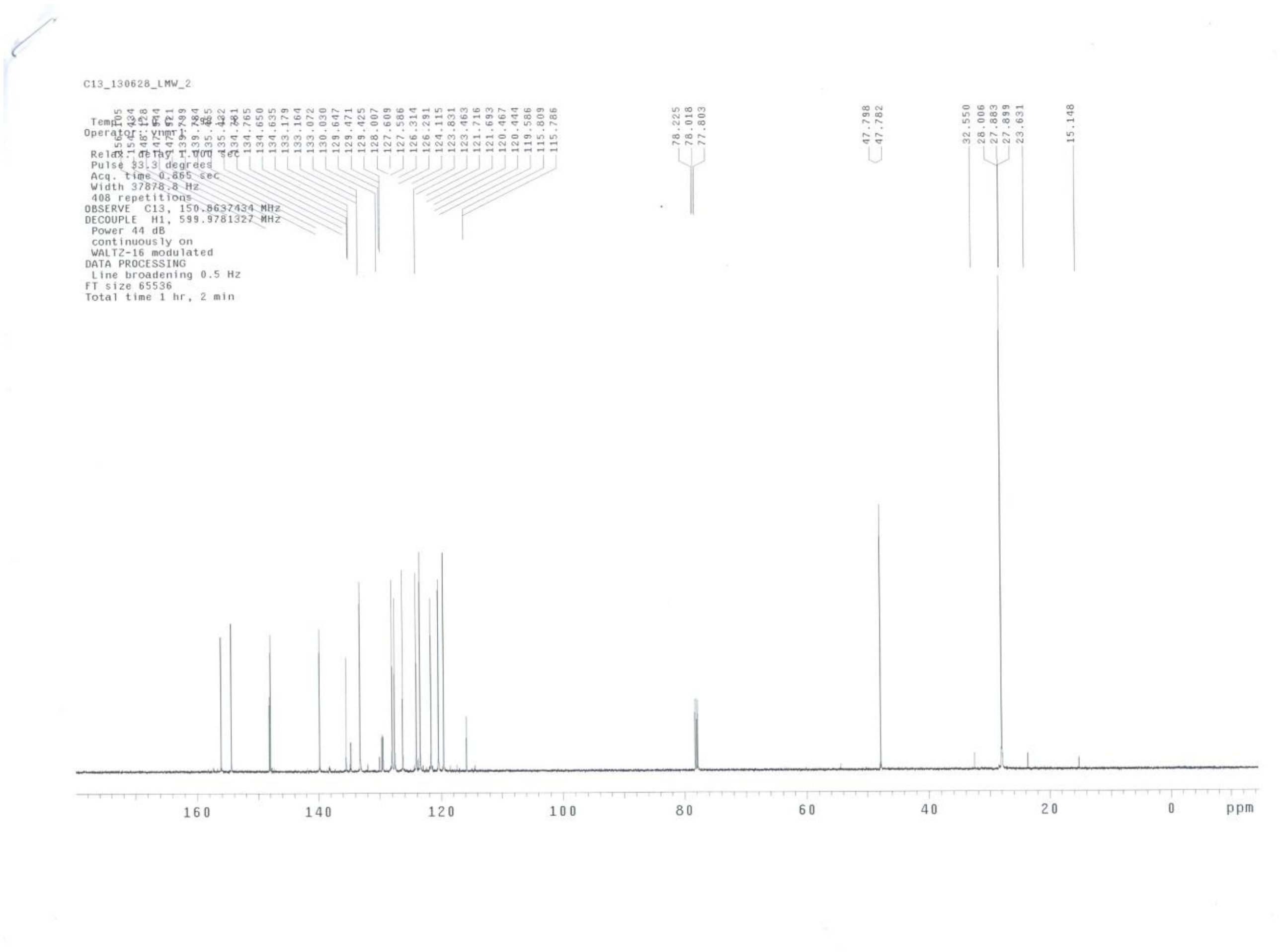

Figure S2 | ^{13}C NMR spectrum of (2).

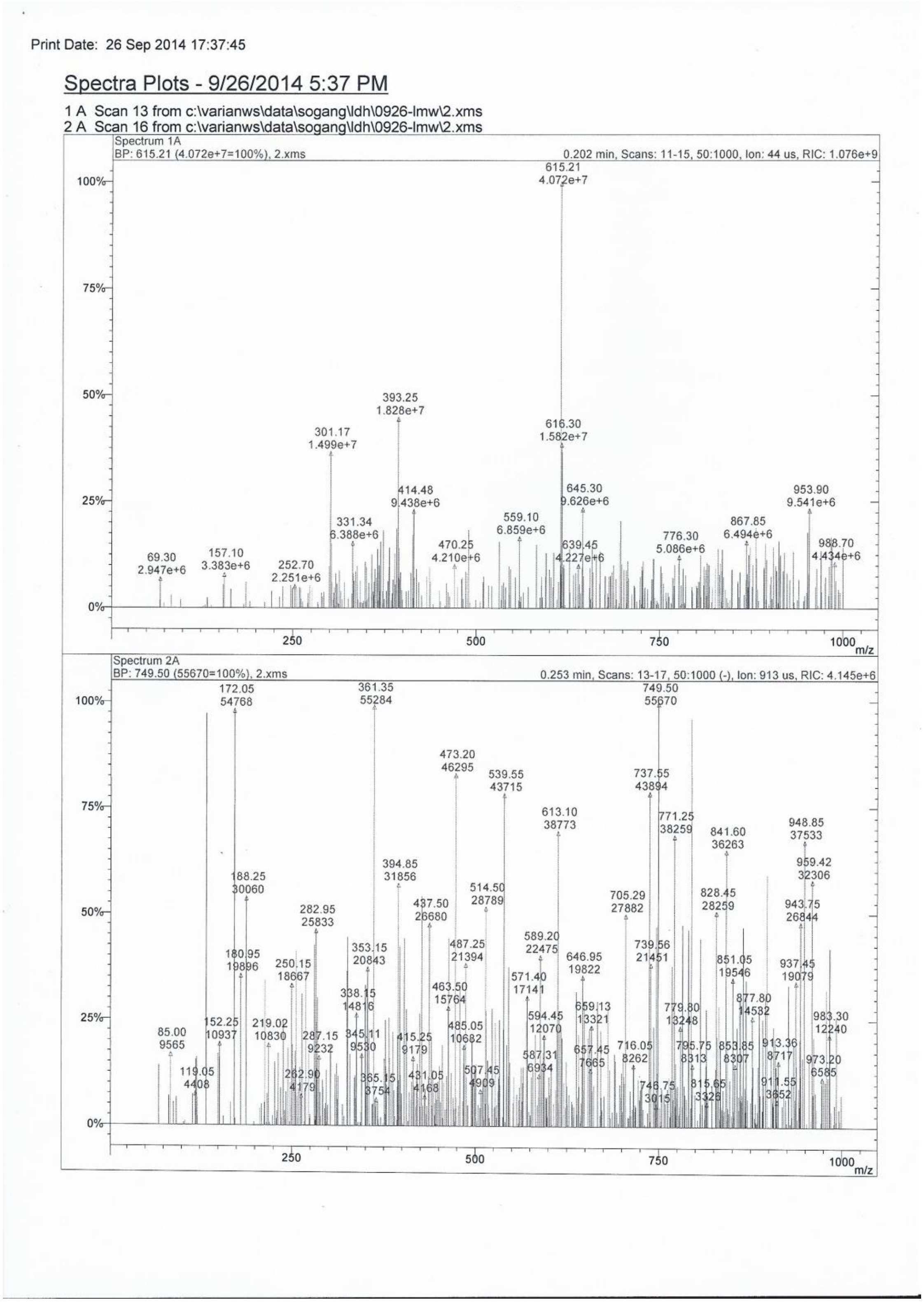

Figure S3 | Low resolution mass spectrum (LRMS) of (2).

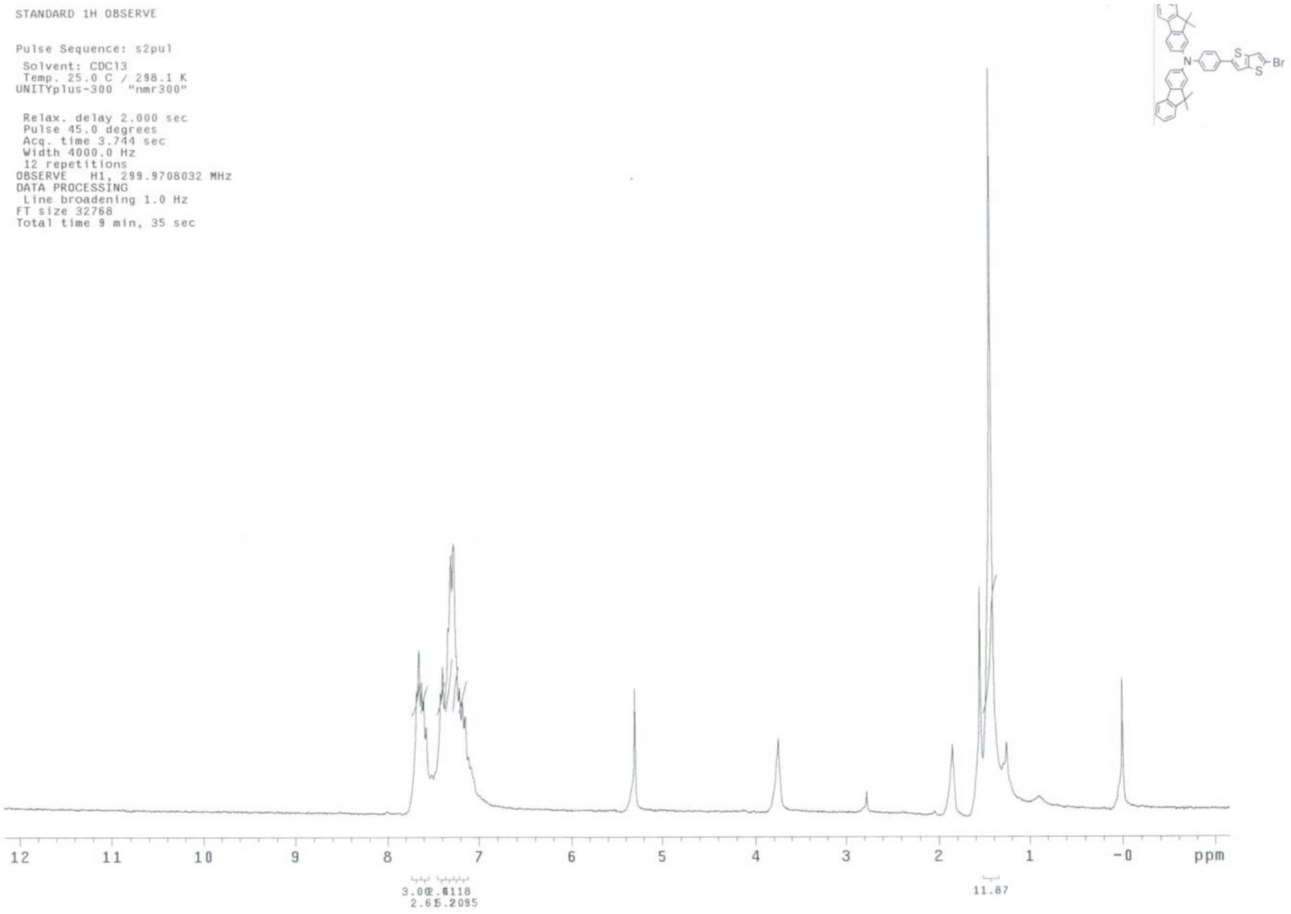

Figure S4 | ^{1}H NMR spectrum of (3).

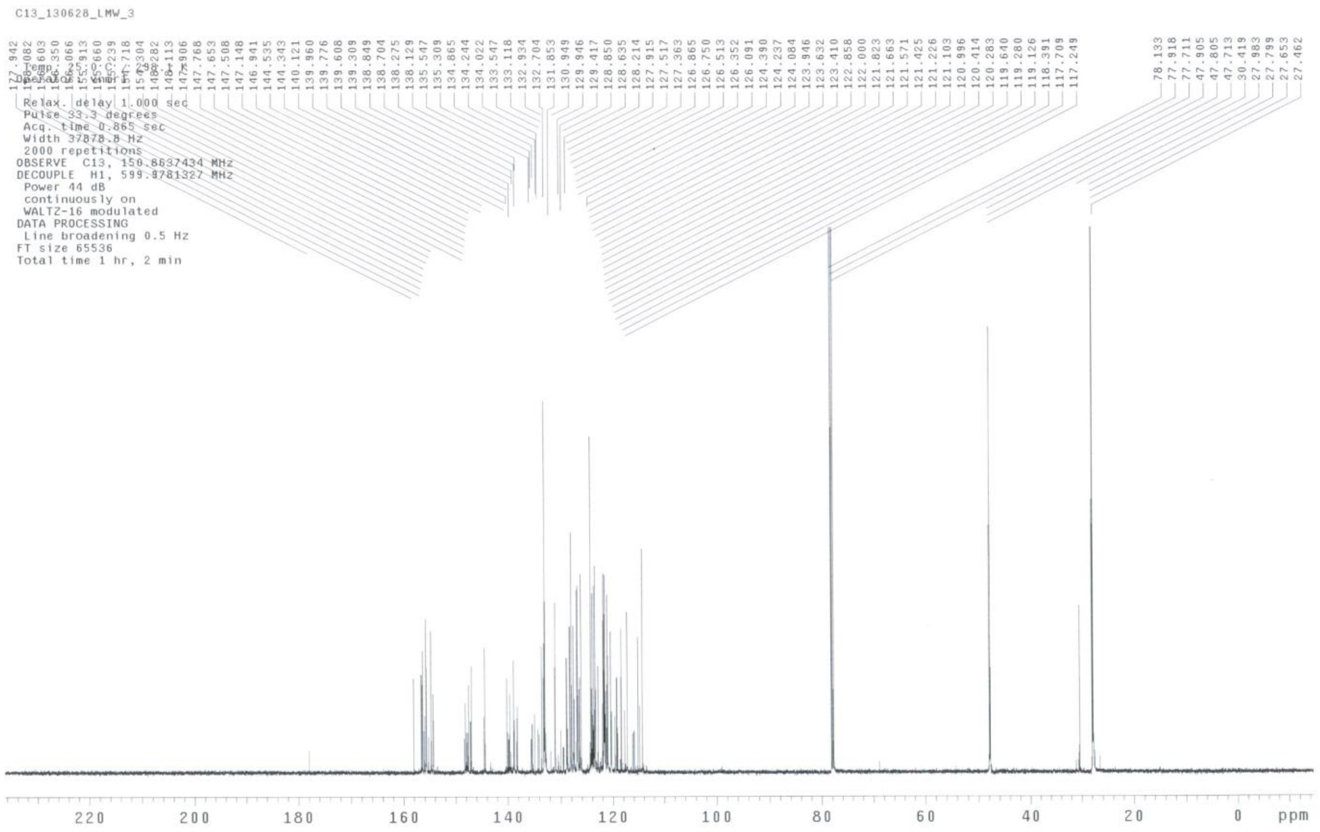

Figure S5 | ^{13}C NMR spectrum of (3).

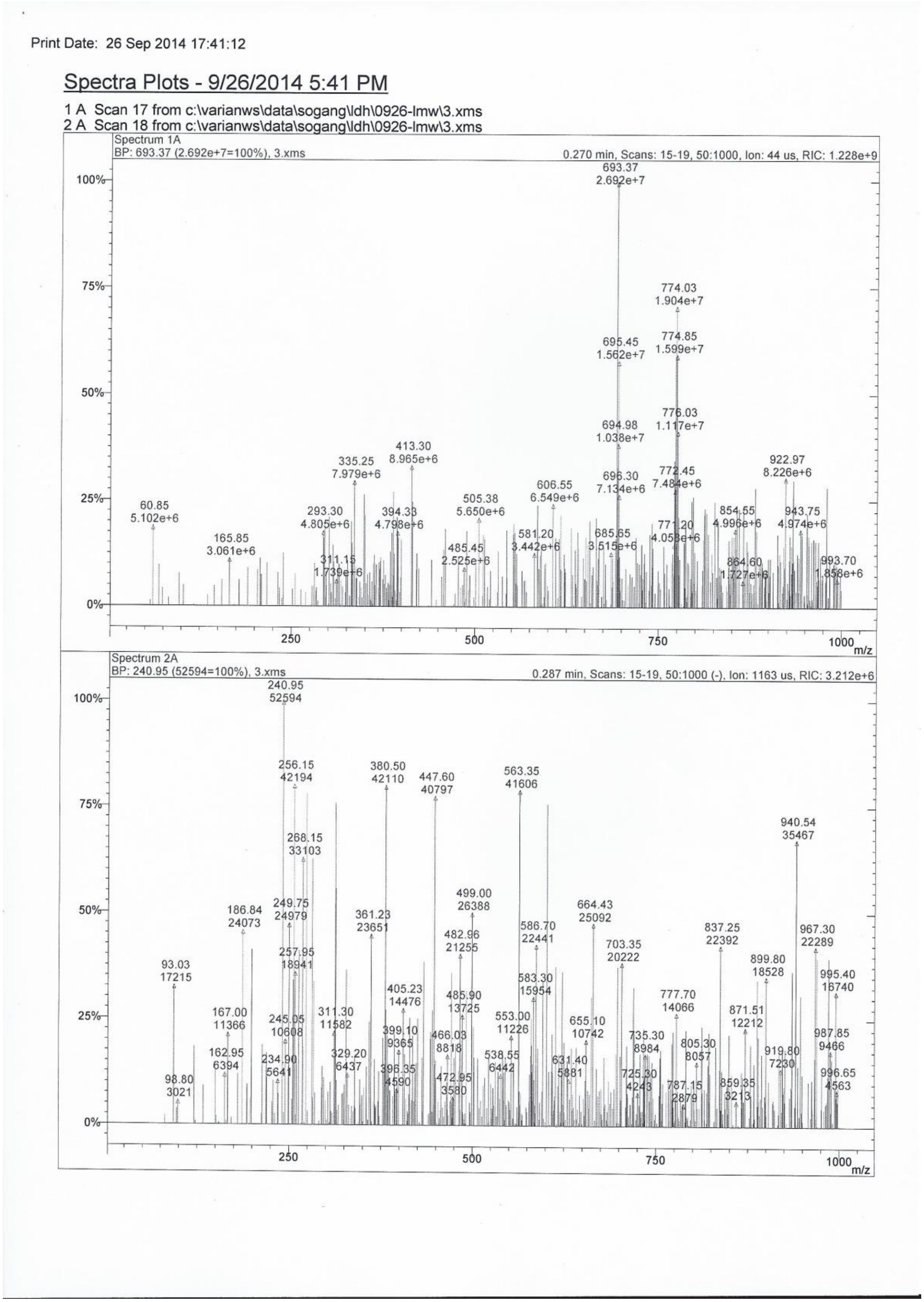

Figure S6 | Low resolution mass spectrum (LRMS) of (3).

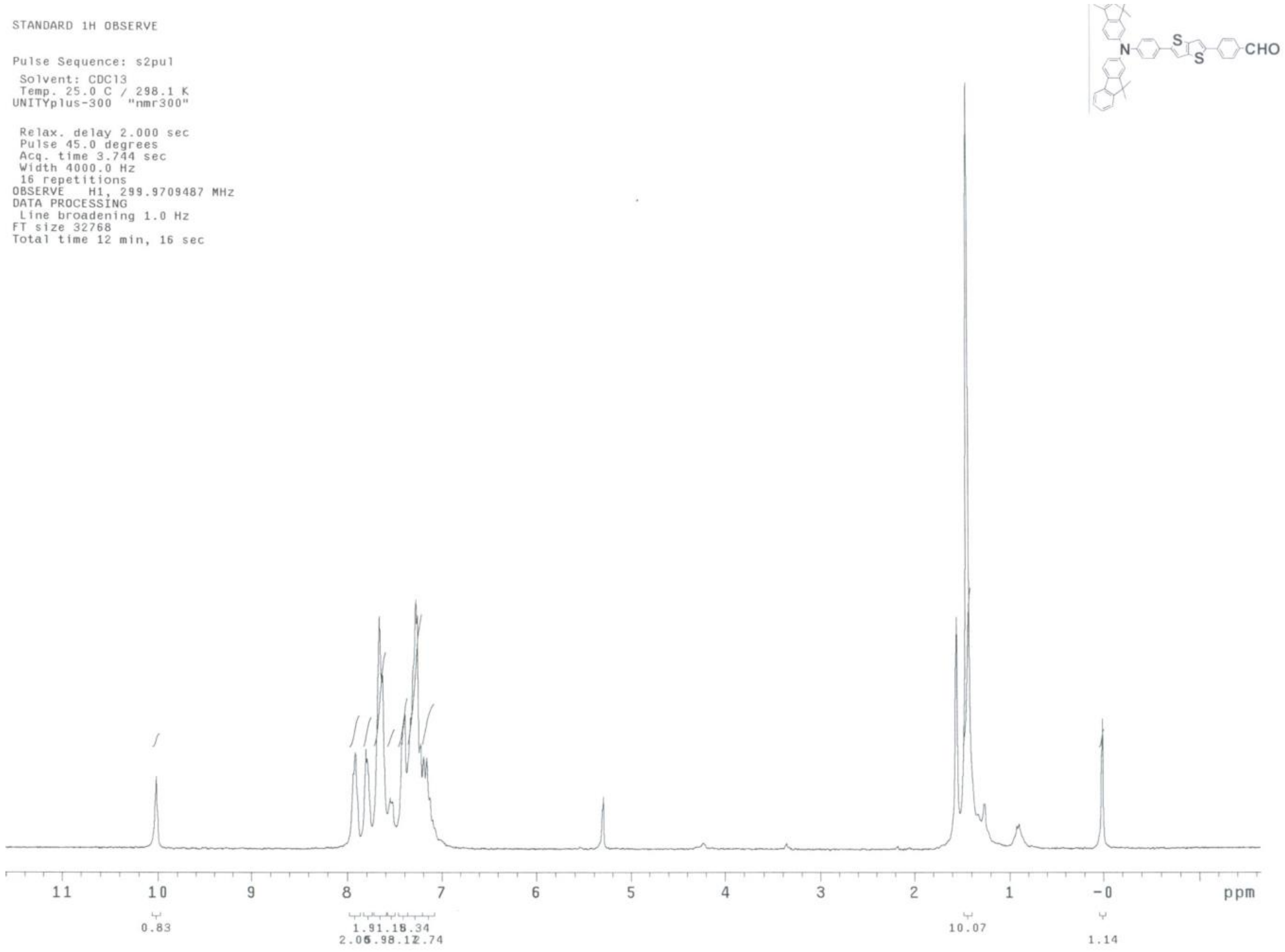

Figure S7 | ^{1}H NMR spectrum of (4).

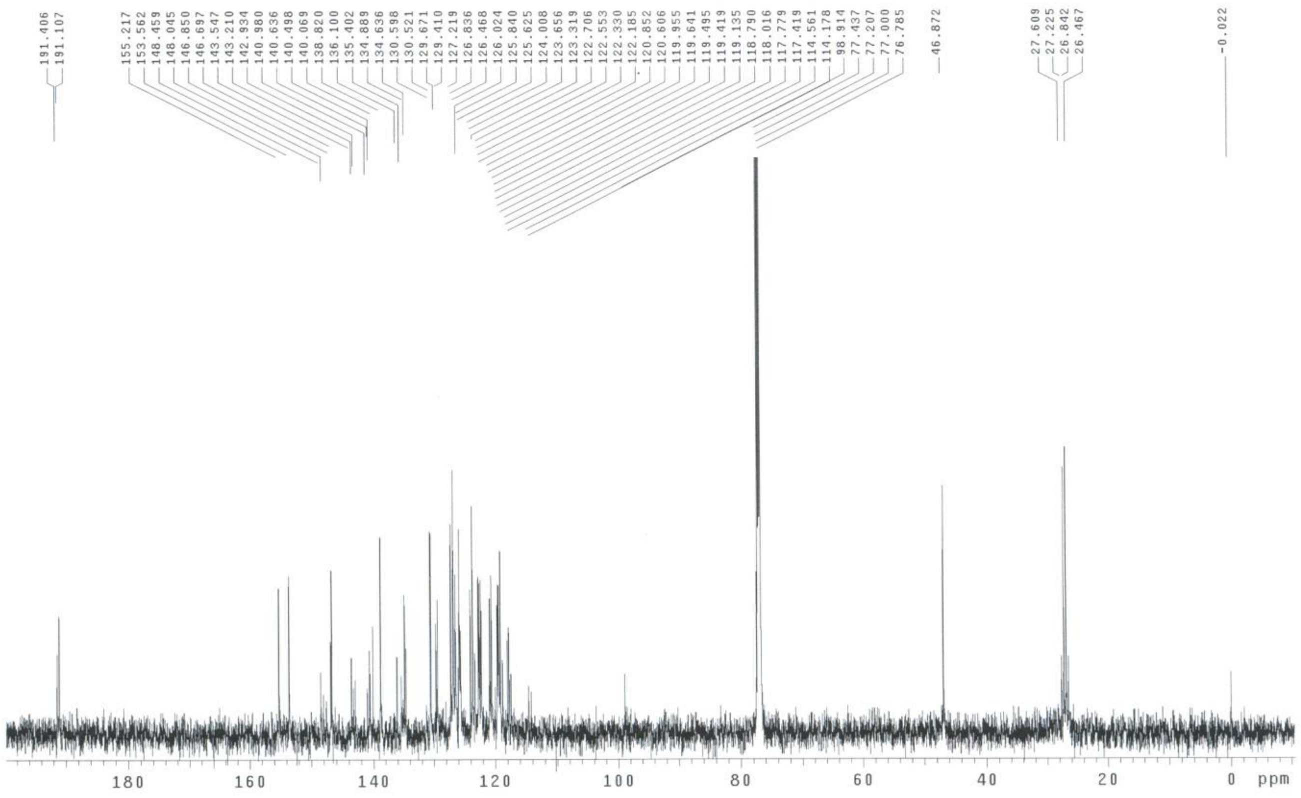

Figure S8 | ^{13}C NMR spectrum of (4).

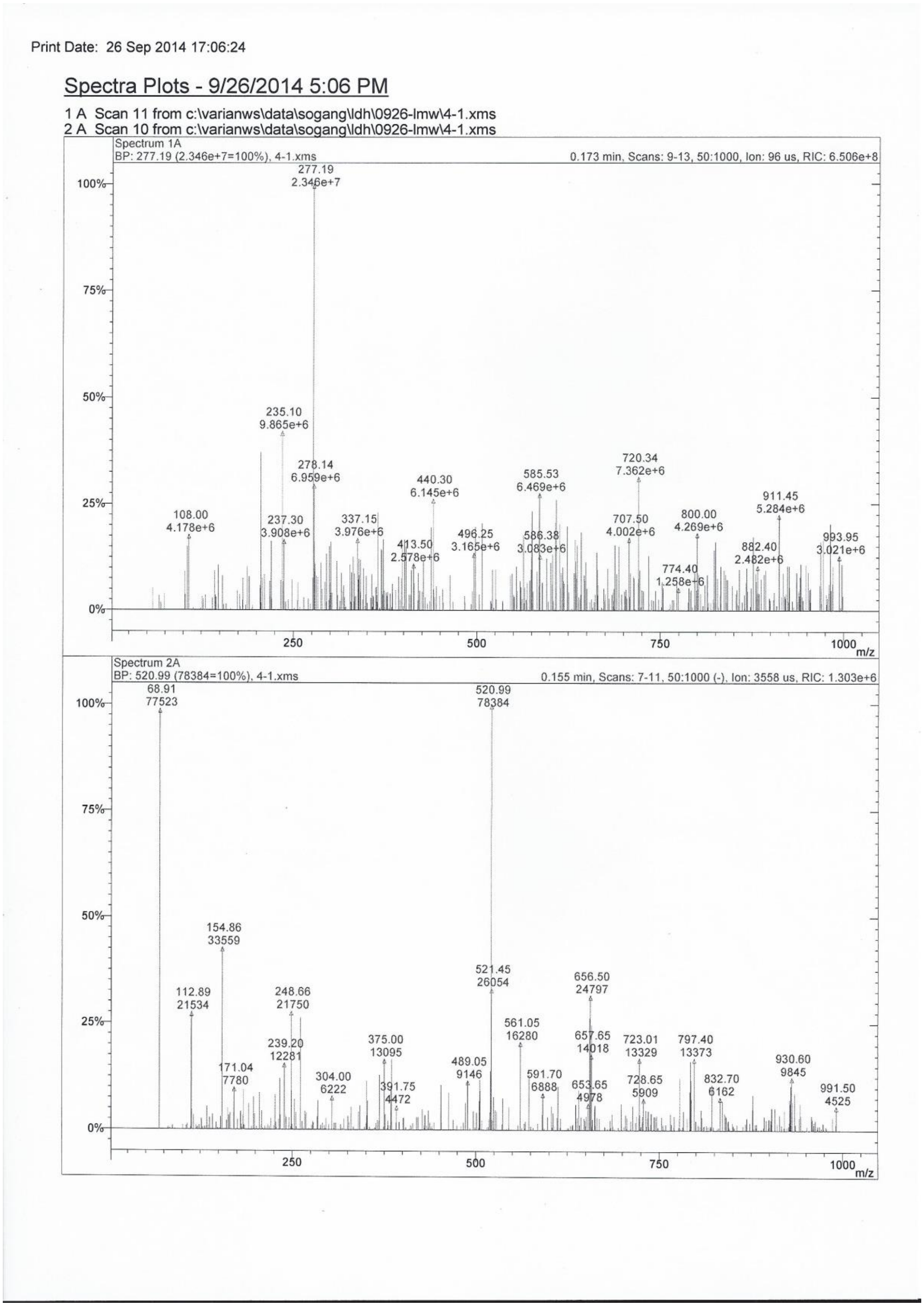

Figure S9 | Low resolution mass spectrum (LRMS) of (4).

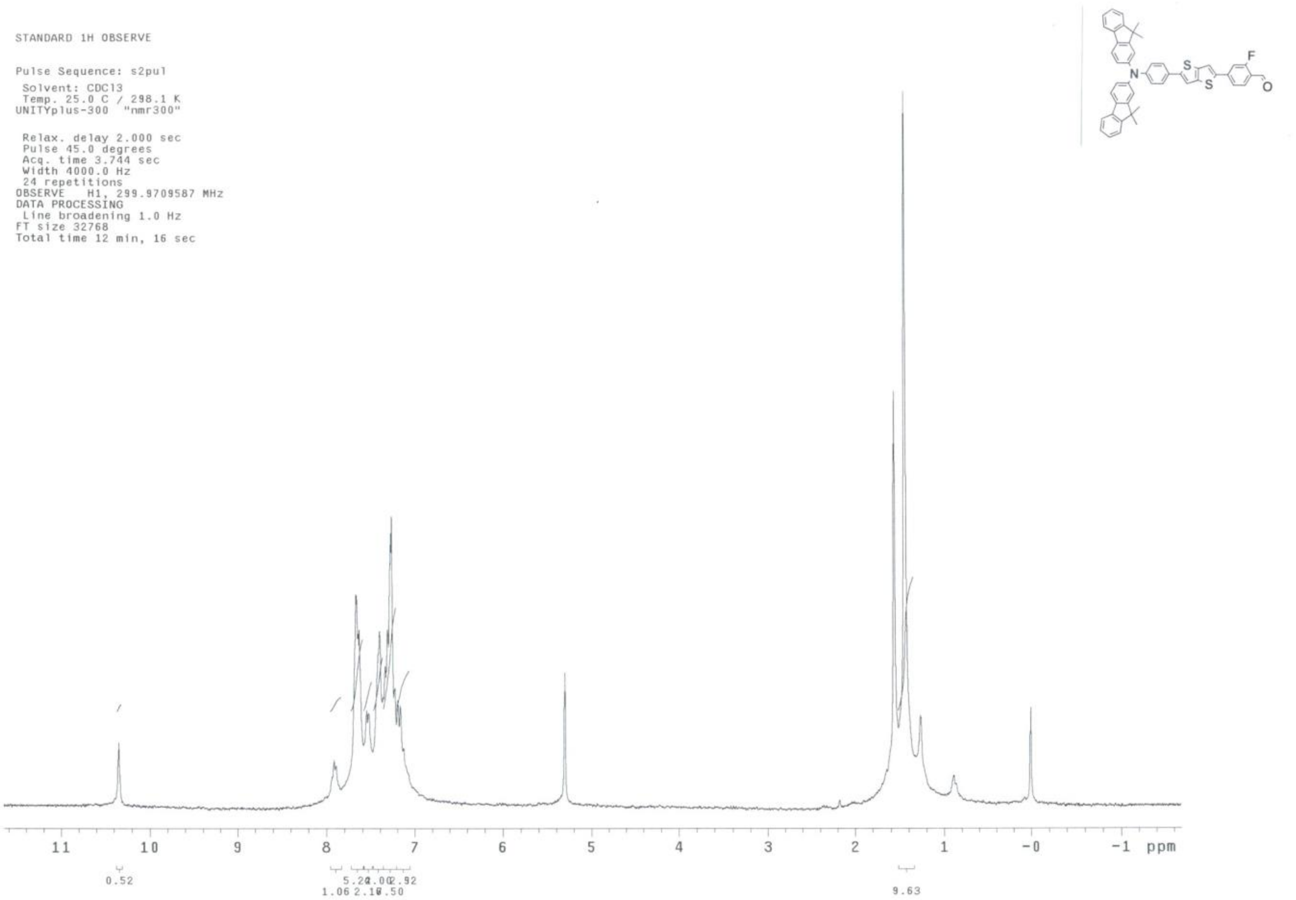

Figure S10 | ^{1}H NMR spectrum of (5).

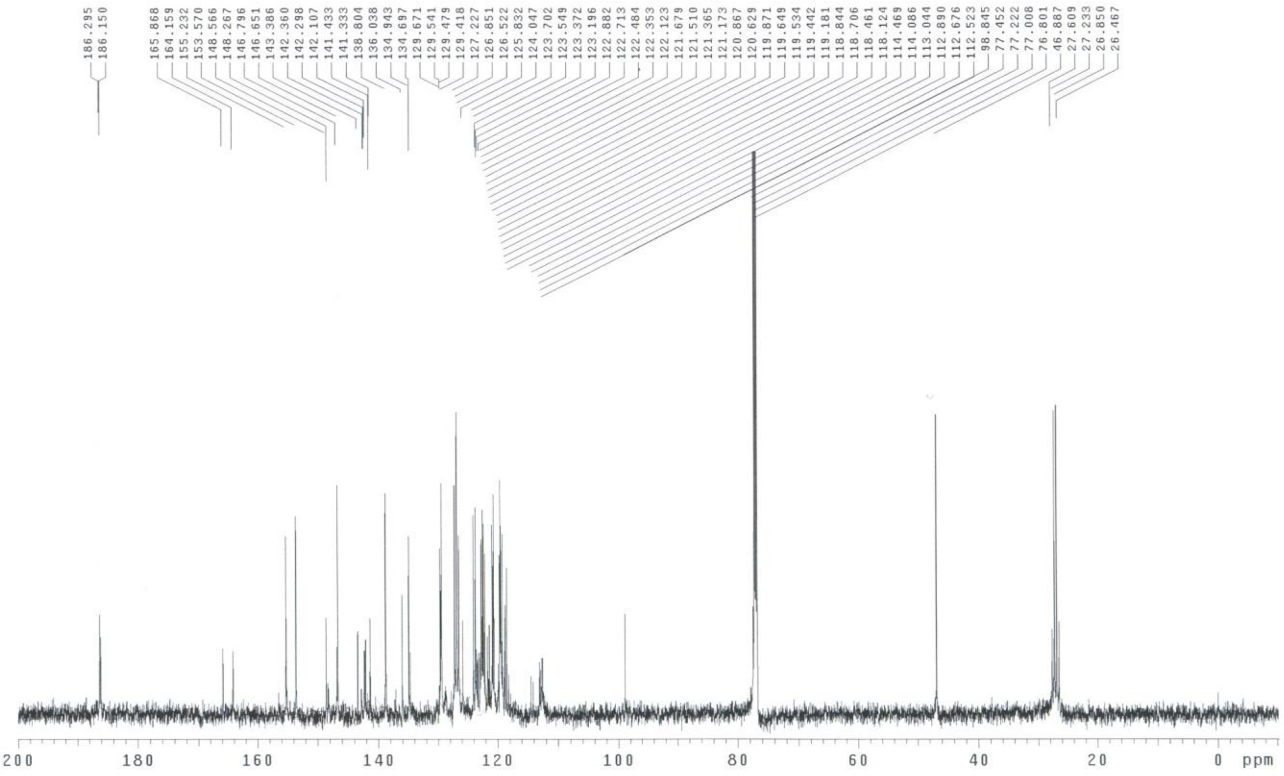

Figure S11 | ^{13}C NMR spectrum of (5).

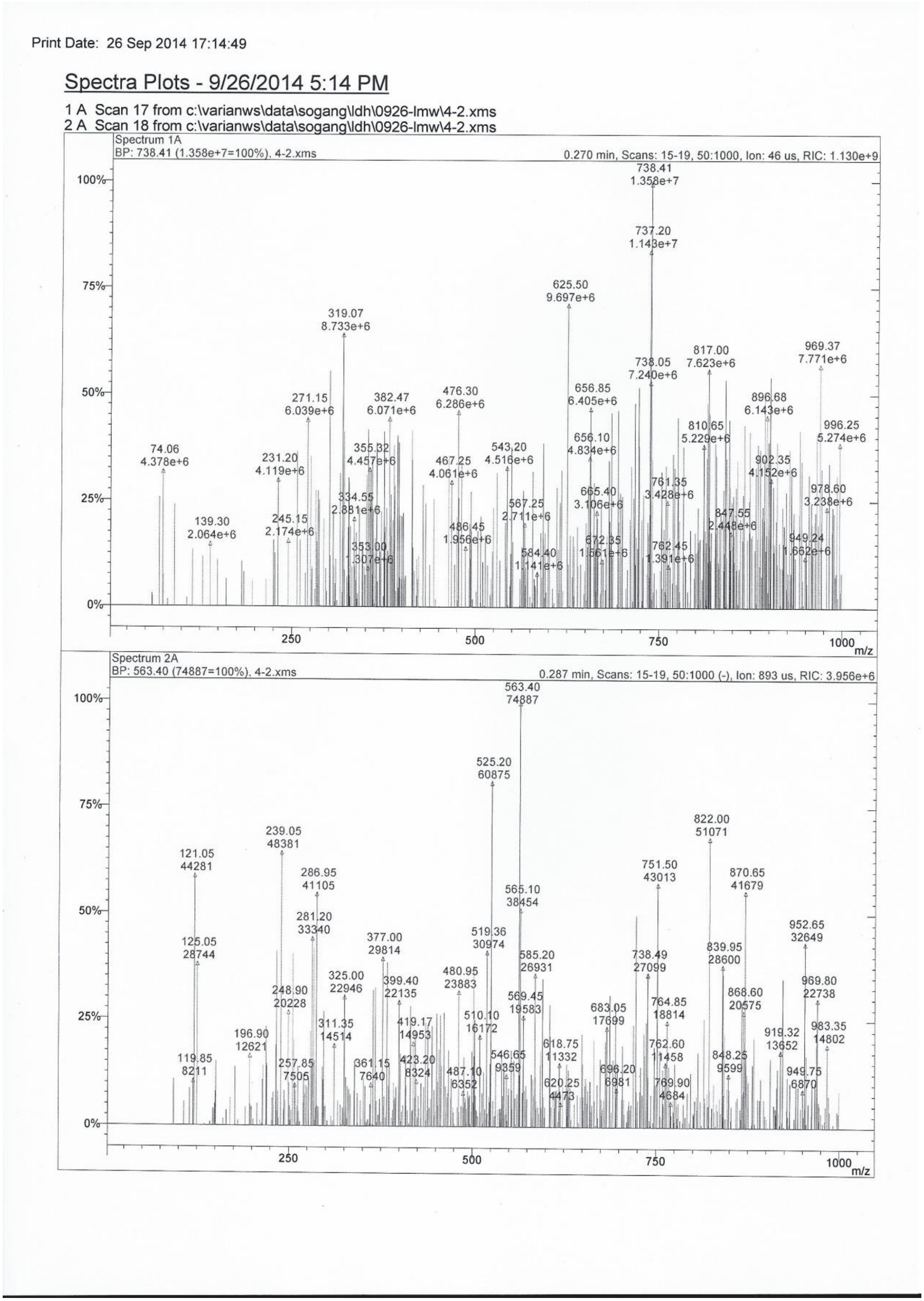

Figure S12 | Low resolution mass spectrum (LRMS) of (5).

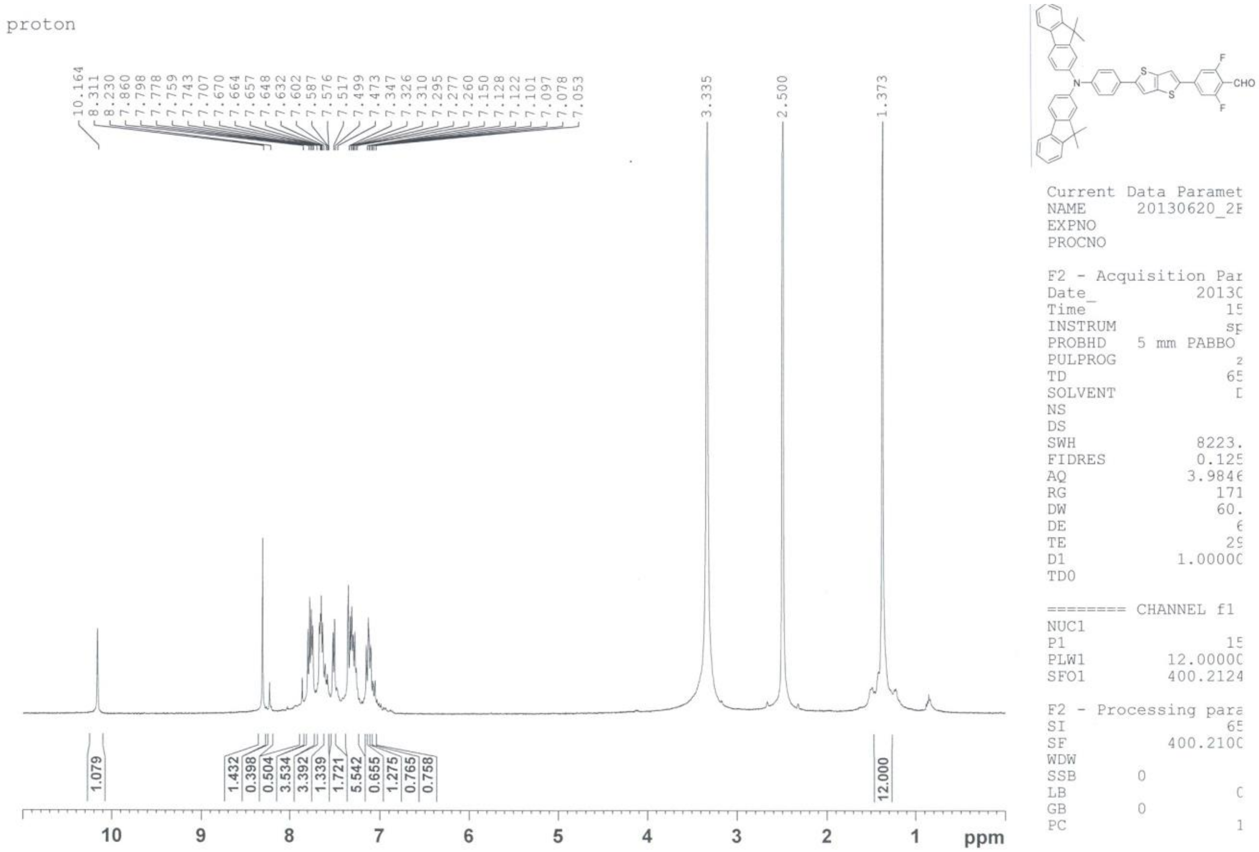

Figure S13 | ^{1}H NMR spectrum of (6).

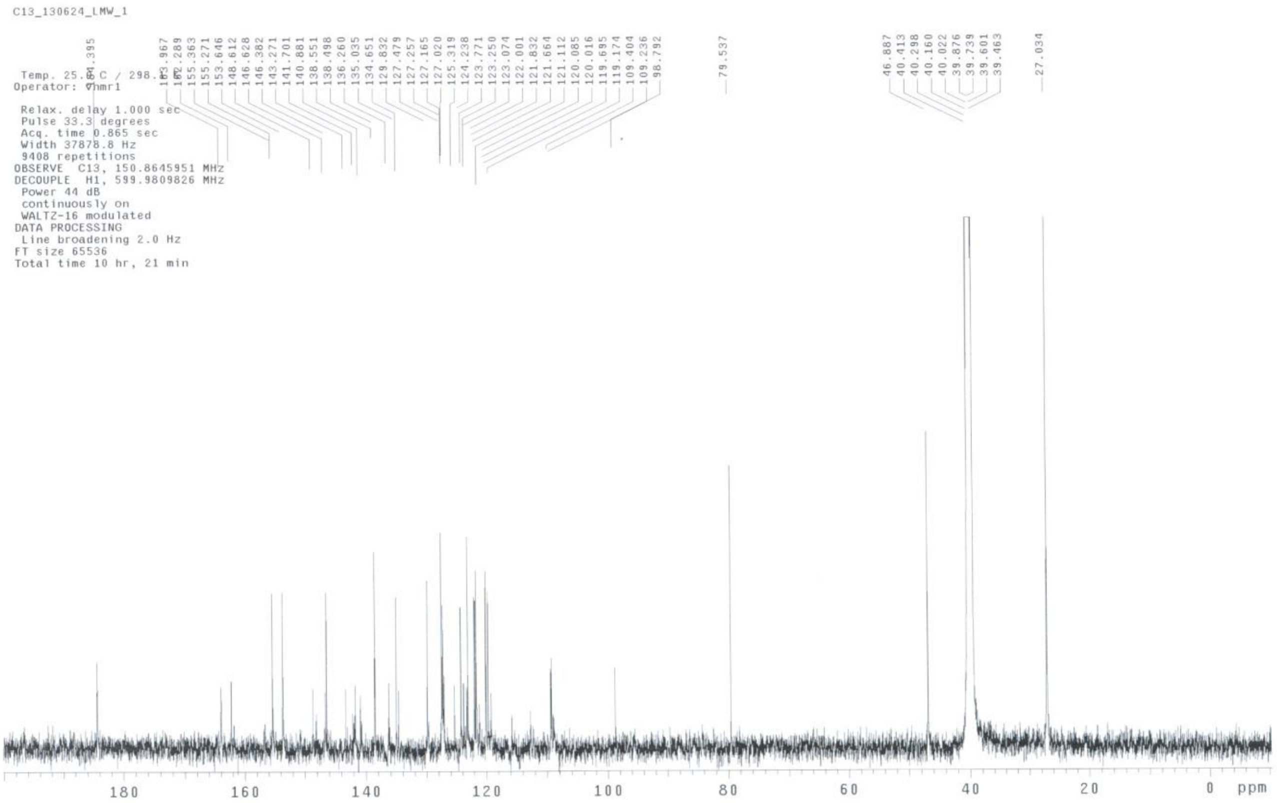

Figure S14 | ^{13}C NMR spectrum of (6).

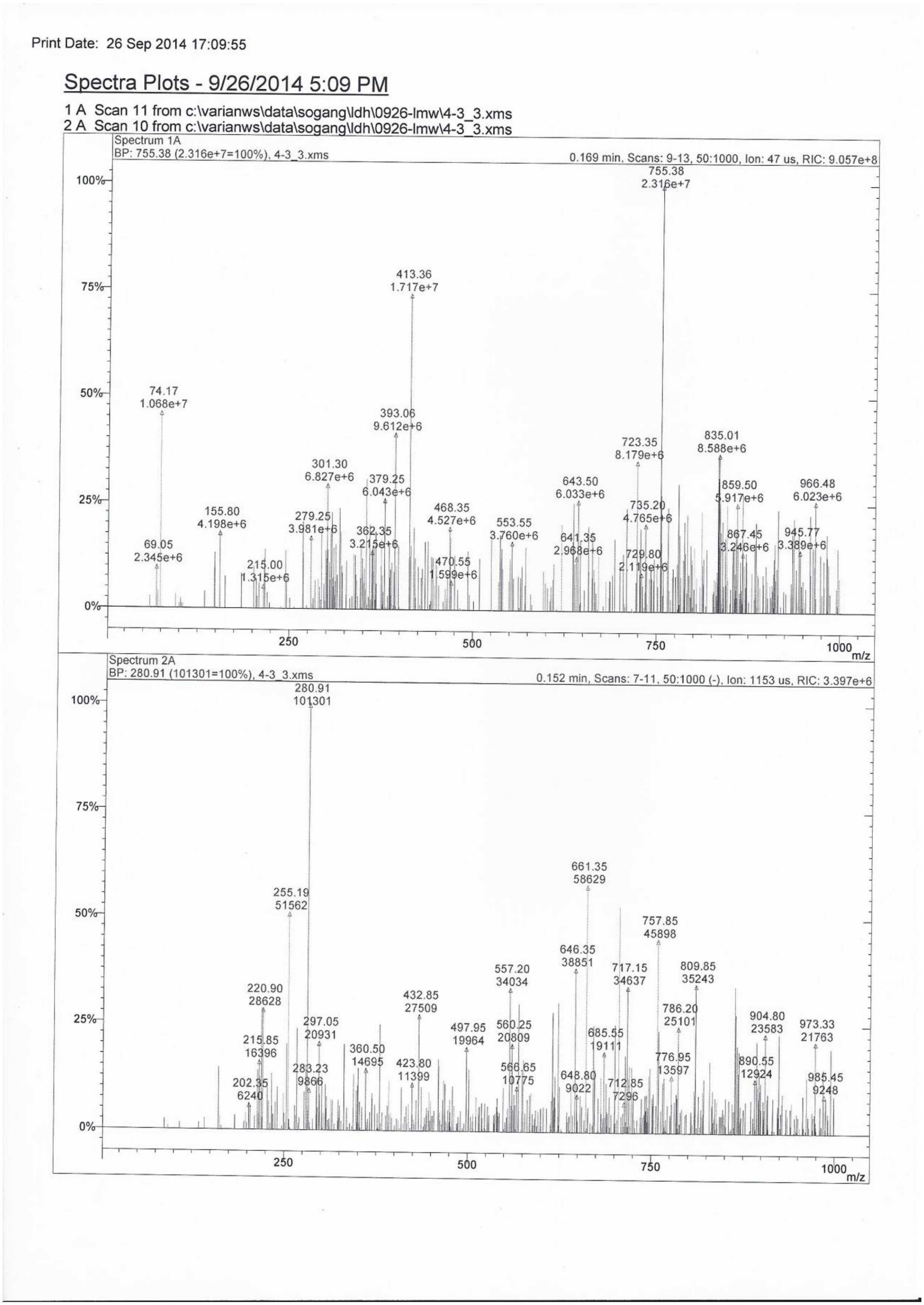

Figure S15 | Low resolution mass spectrum (LRMS) of (6).

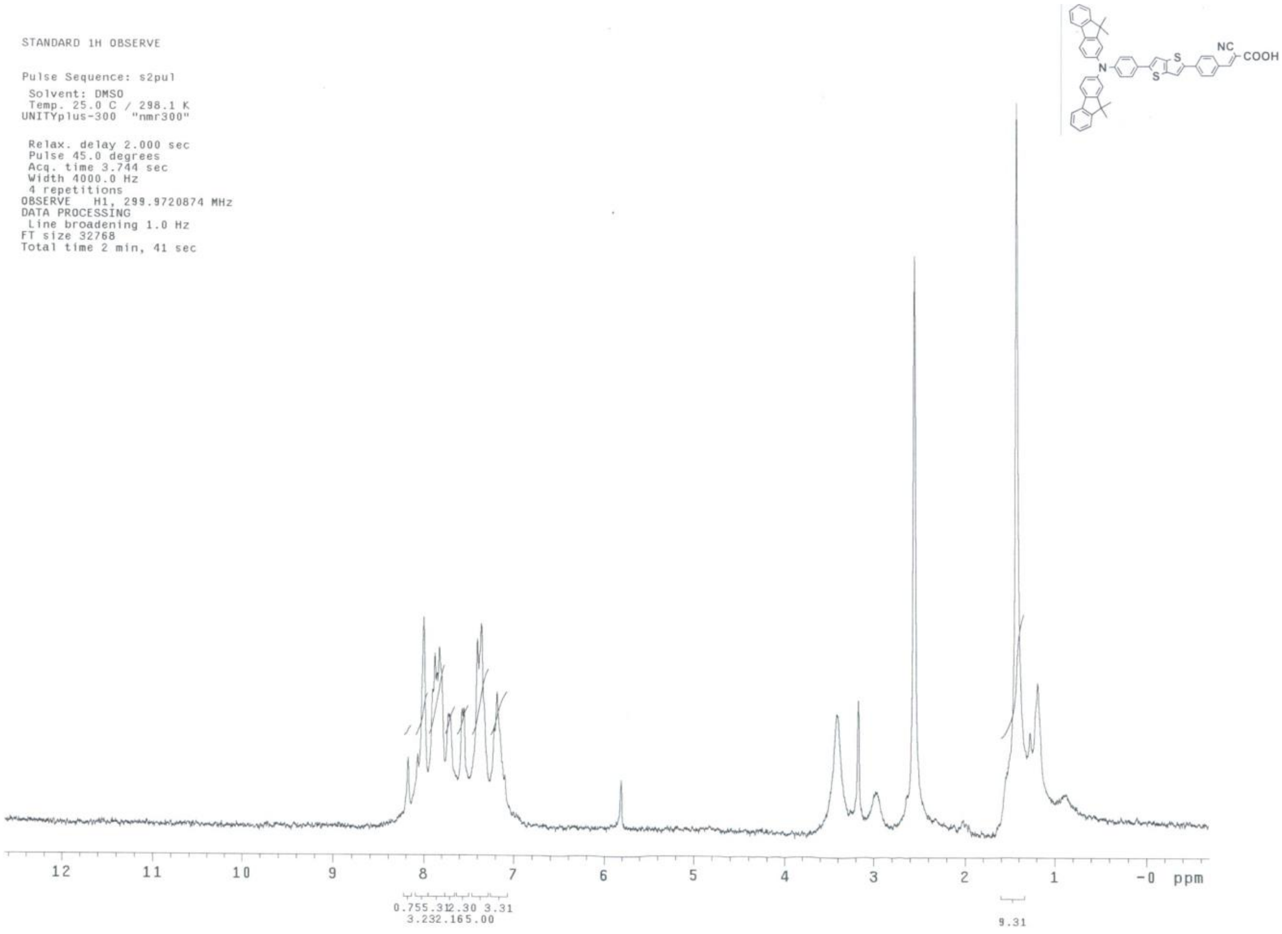

Figure S16 | ^{1}H NMR spectrum of M5.

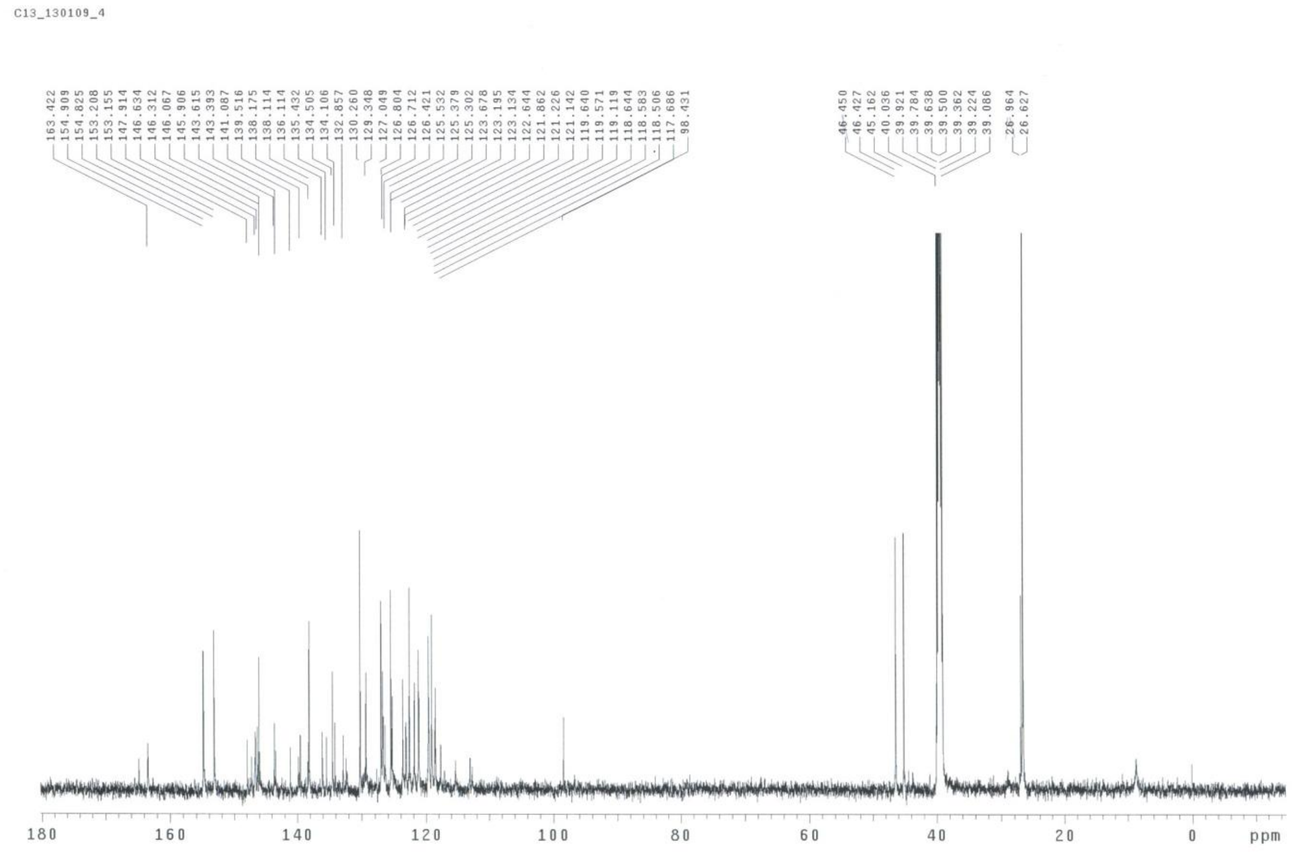

Figure S17 | ^{13}C NMR spectrum of M5.

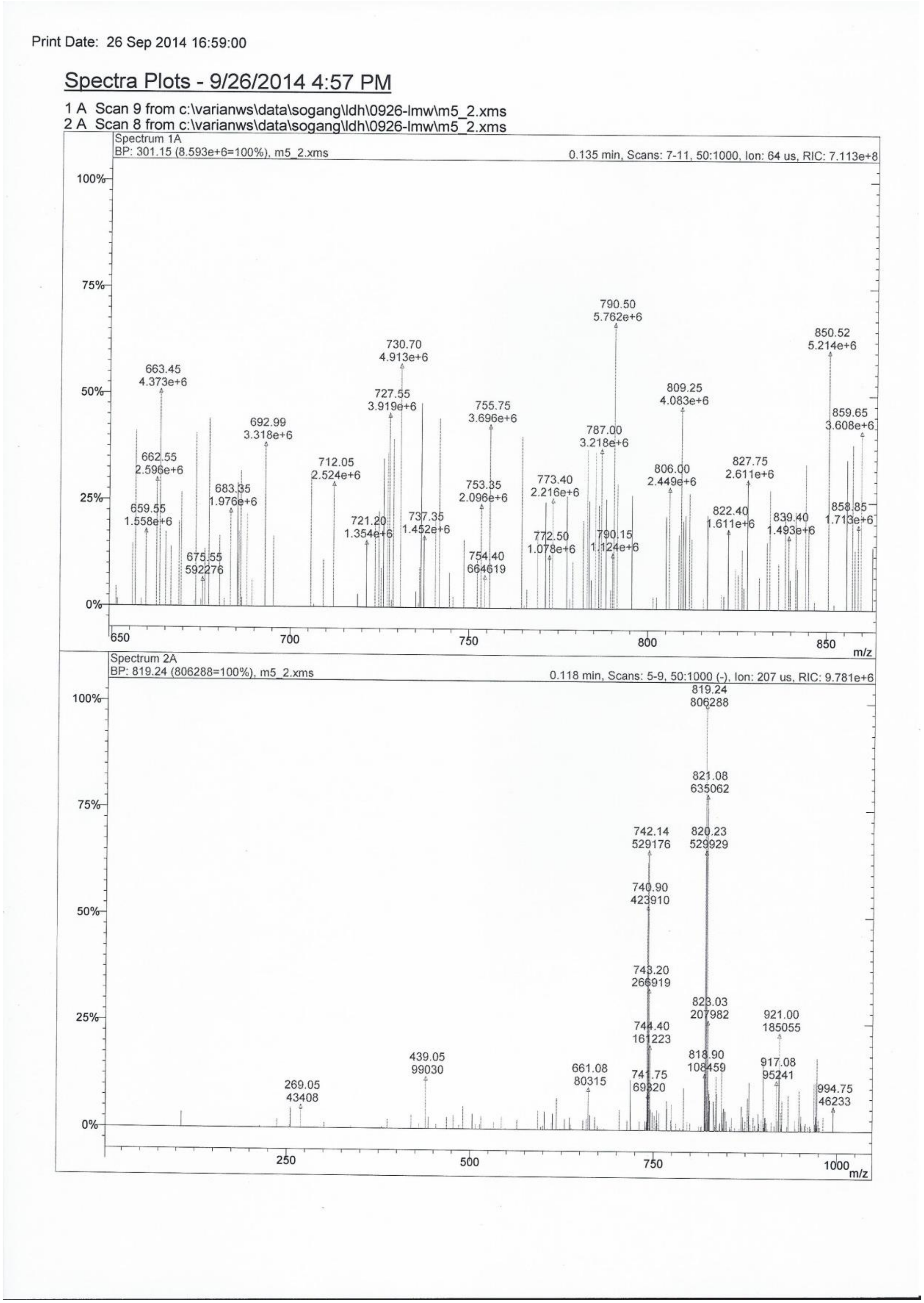

Figure S18 | Low resolution mass spectrum (LRMS) of M5.

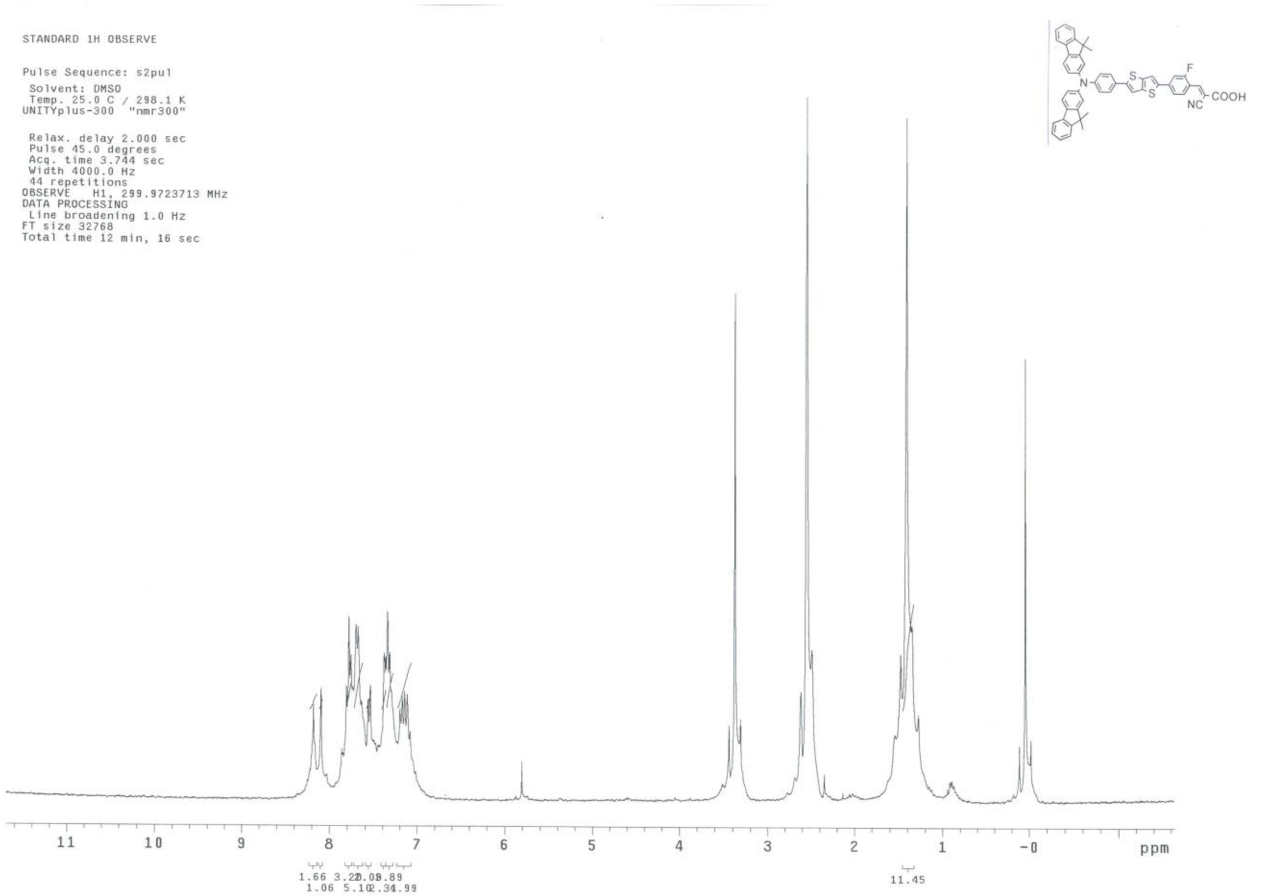

Figure S19 | ^{1}H NMR spectrum of M6.

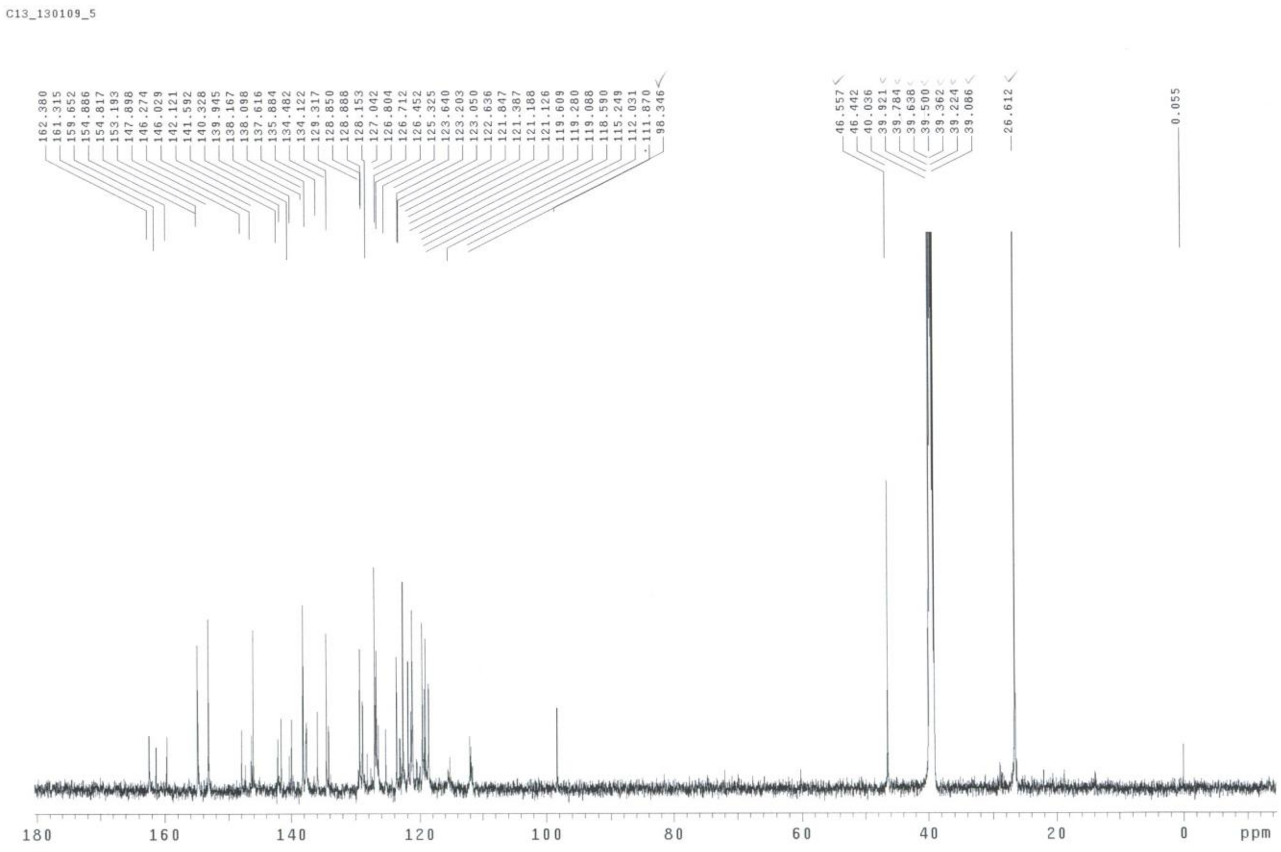

Figure S20 | ^{13}C NMR spectrum of M6.

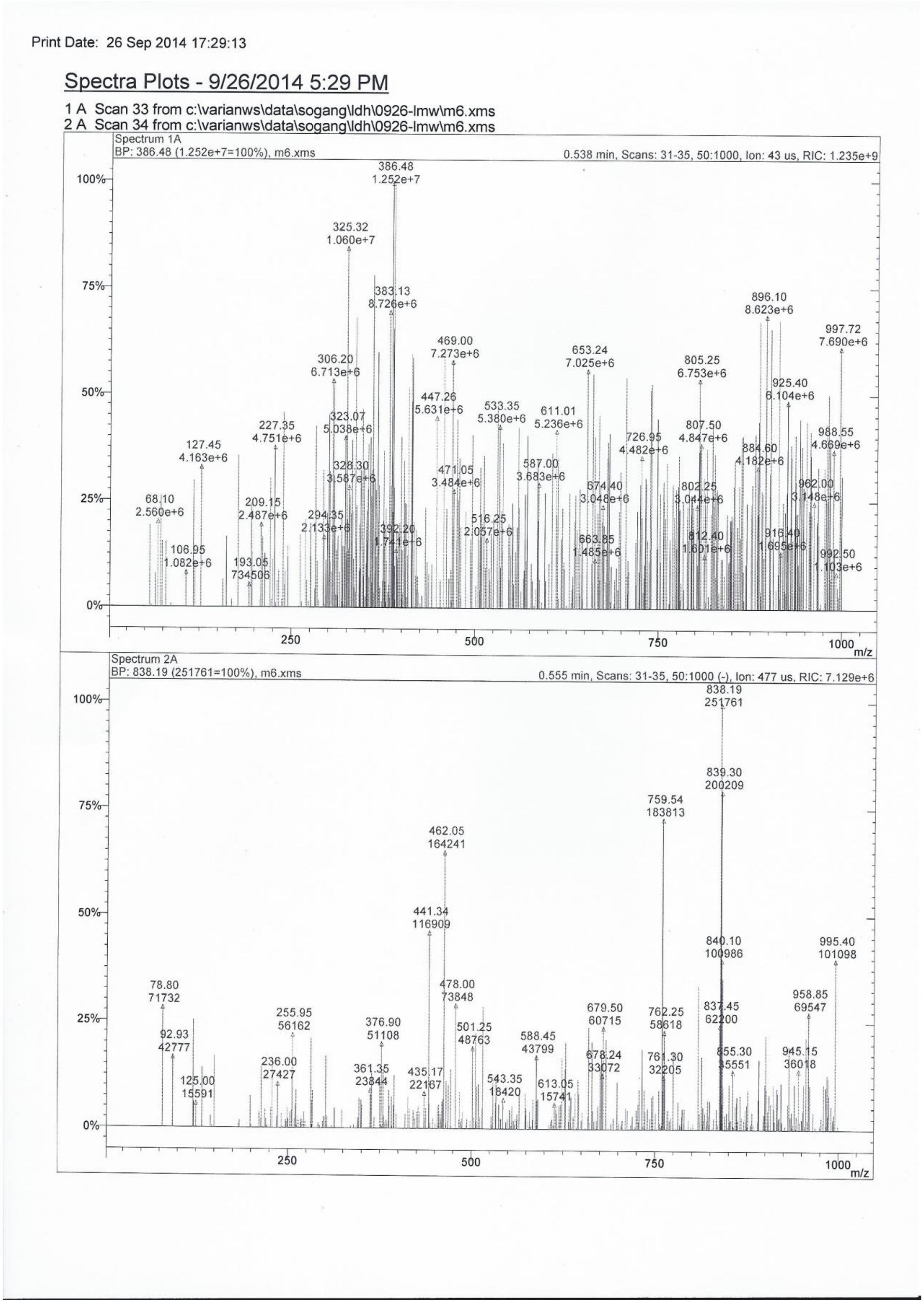

Figure S21 | Low resolution mass spectrum (LRMS) of M6.

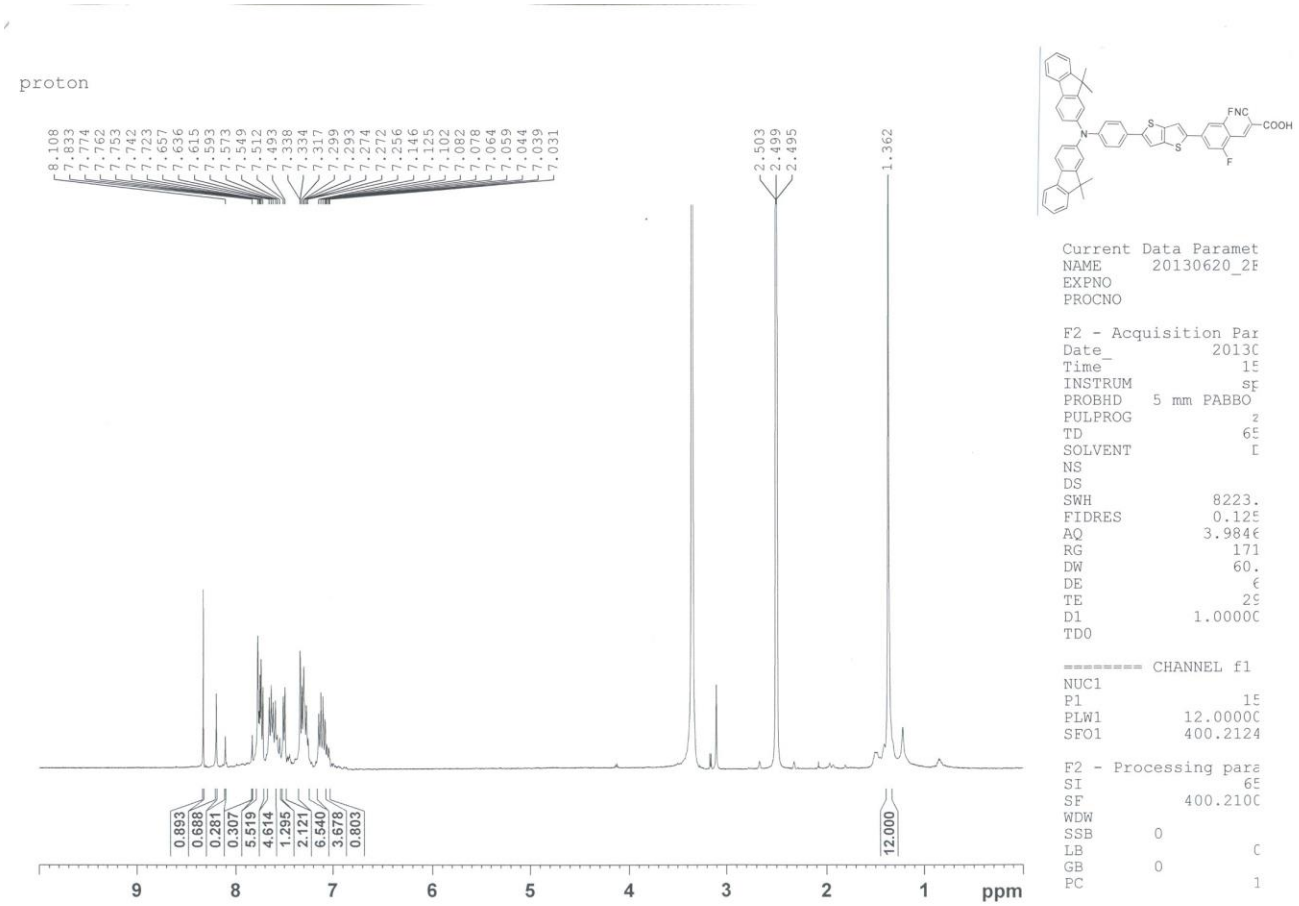

Figure S22 | ^{1}H NMR spectrum of M7.

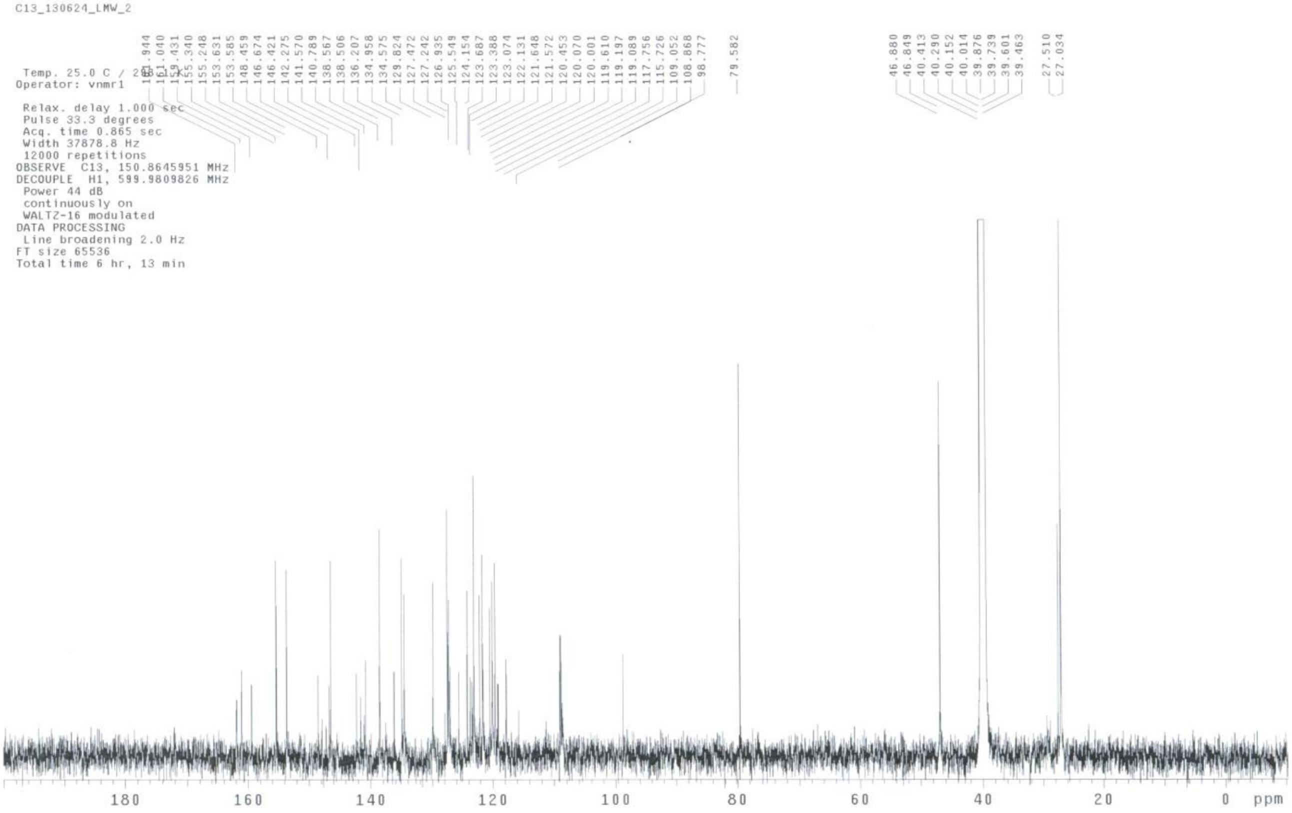

Figure S23 | ^{13}C NMR spectrum of M7.

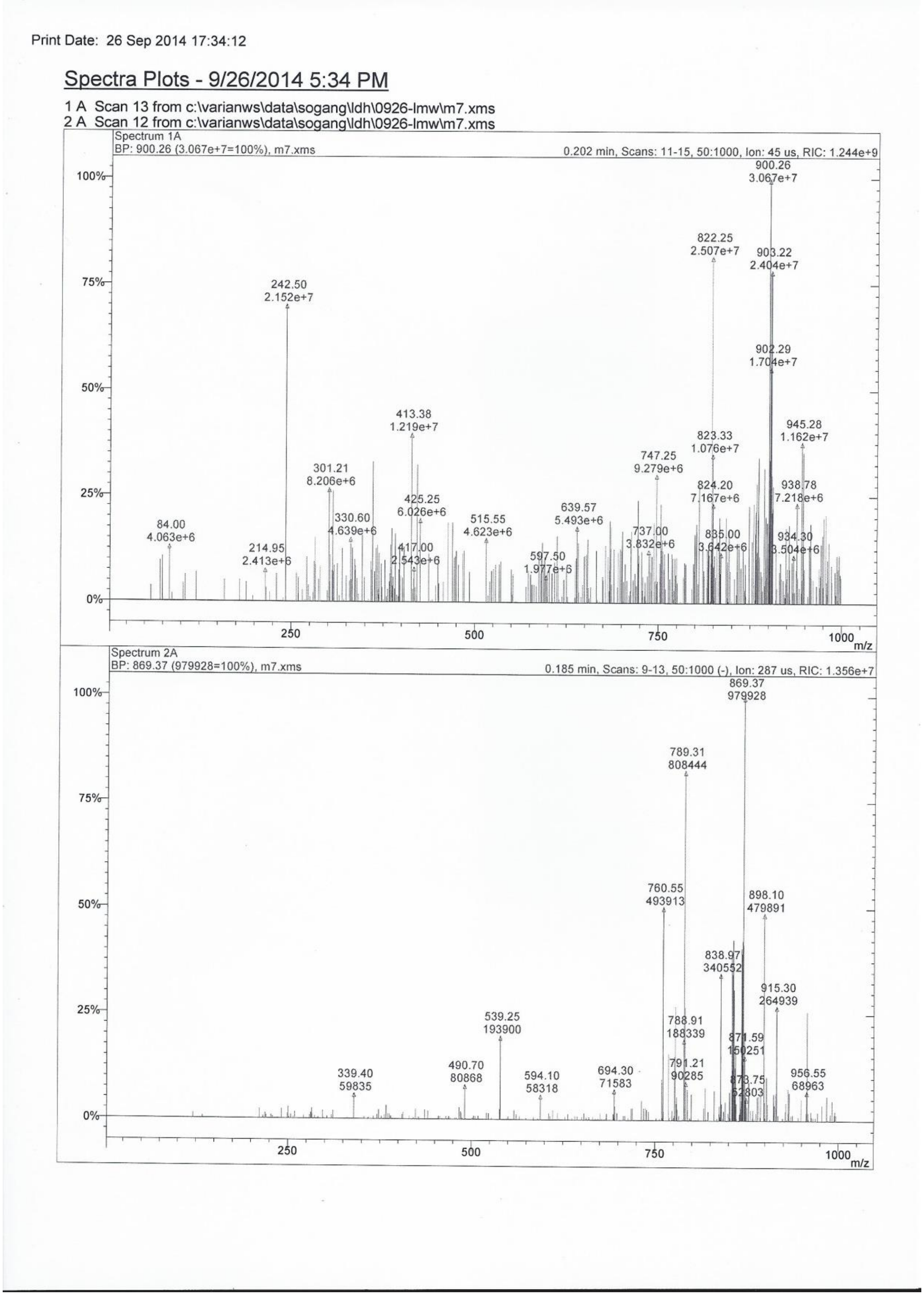

Figure S24 | Low resolution mass spectrum (LRMS) of M7.

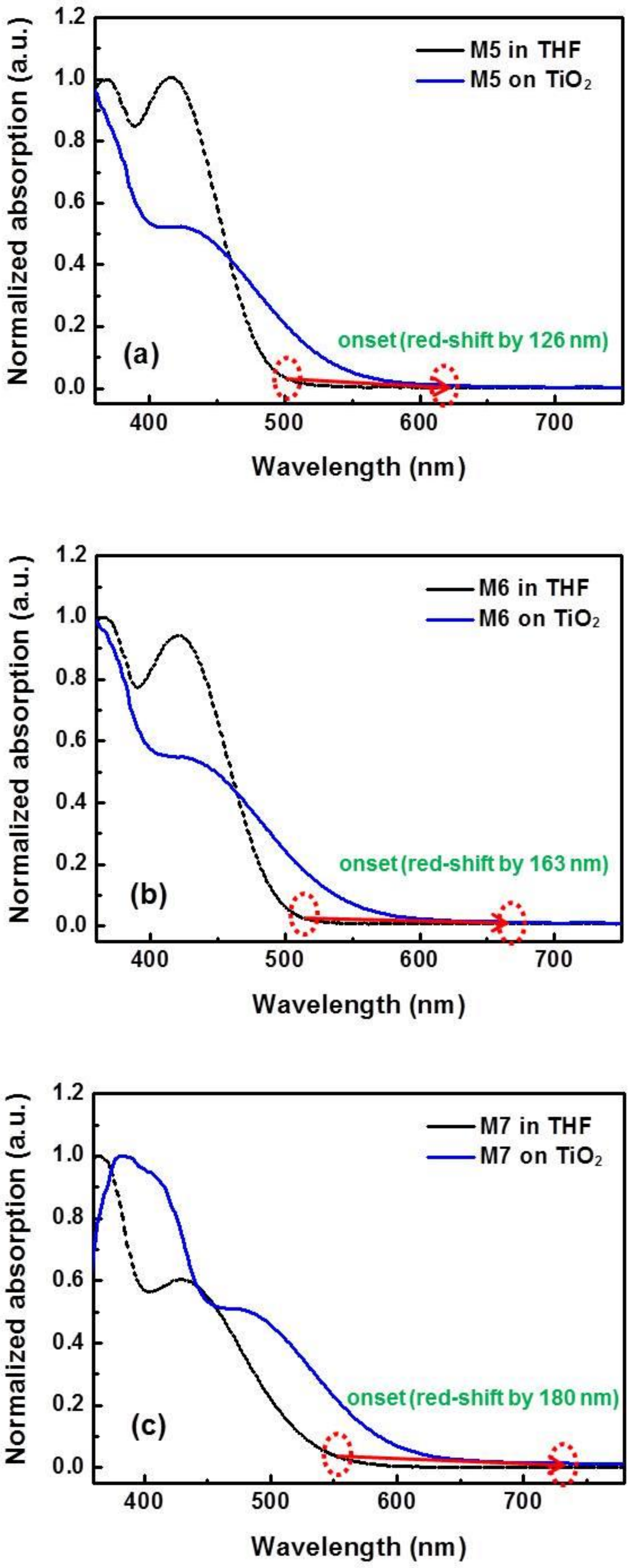

Figure S25 | Comparison of the UV-vis absorption onset measured in THF and on TiO_2 films of the M series.

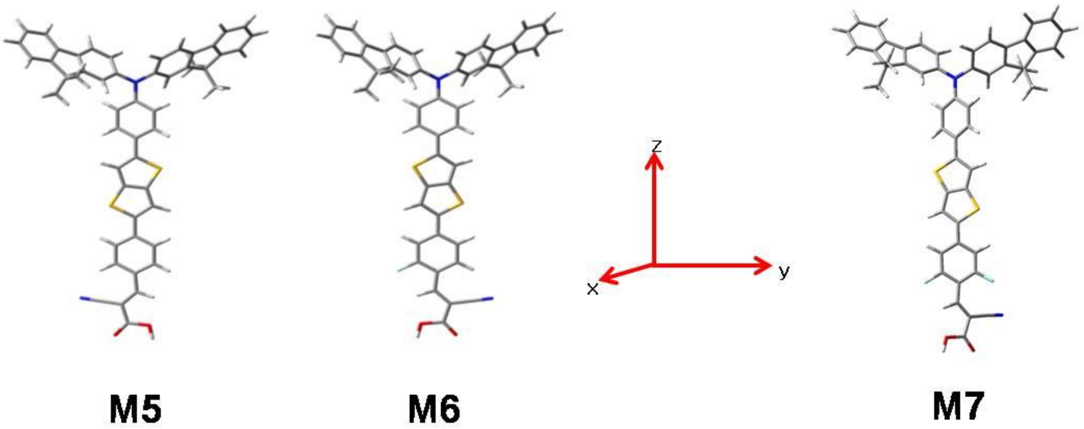

Figure S26 | Optimized geometrical structures of the M series. The reference axes are also shown by the red trihedron. The *z* axis corresponds to the TiO_2 surface normal.

Table S1 | Calculated dipole components of the M series.[a]

Dye	Dipole components (Debye)		
	μ_x	μ_y	μ_z
M5	–2.8	–3.9	6.0
M6	–1.9	–3.1	7.2
M7	–4.2	–1.9	8.5

[a] The *z* axis corresponds to the TiO_2 surface normal.

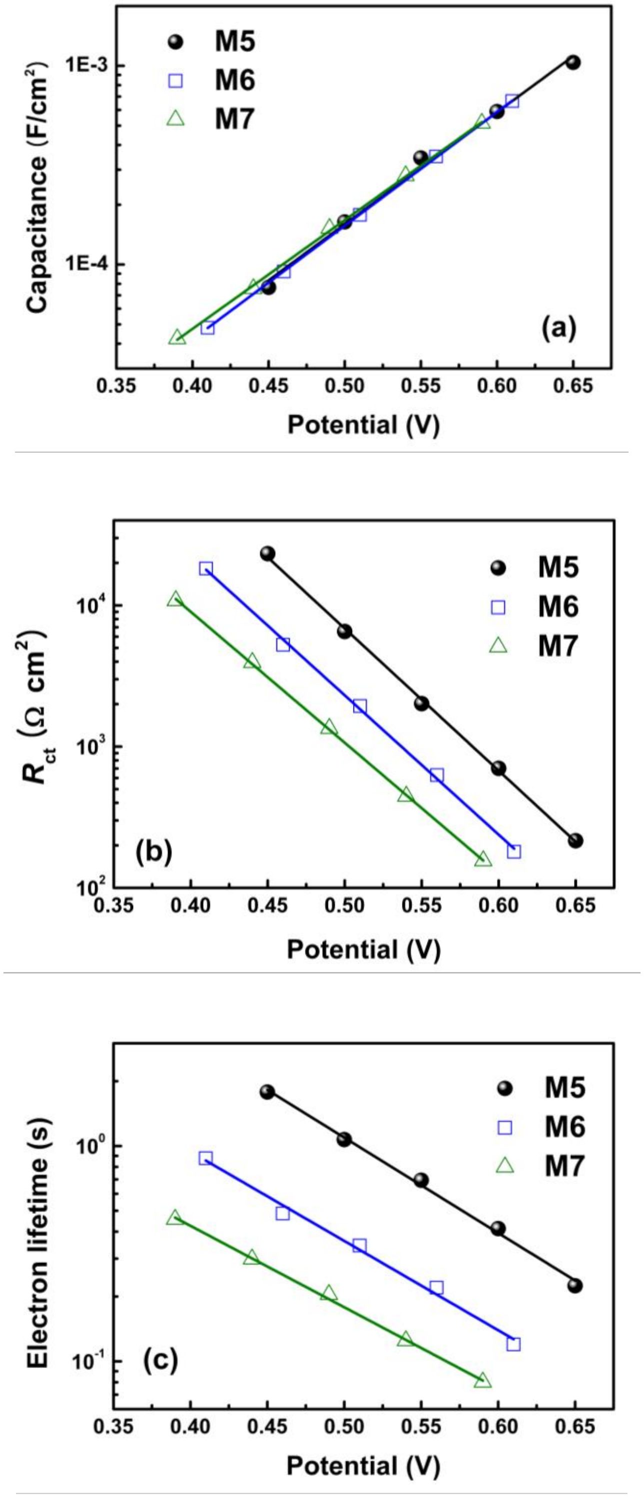

Figure S27 | Electrochemical impedance spectroscopy characterization of DSSCs employing the M series. (a) Chemical capacitance. The plots of **M6** and **M7** were shifted by +40 and +60 mA, respectively, from the original ones to coincide with the plot of **M5**. (b) Charge transfer resistance and (c) electron lifetime with a corresponding potential displacement of +40 and +60 mA for **M6** and **M7**, respectively, from the original plots.

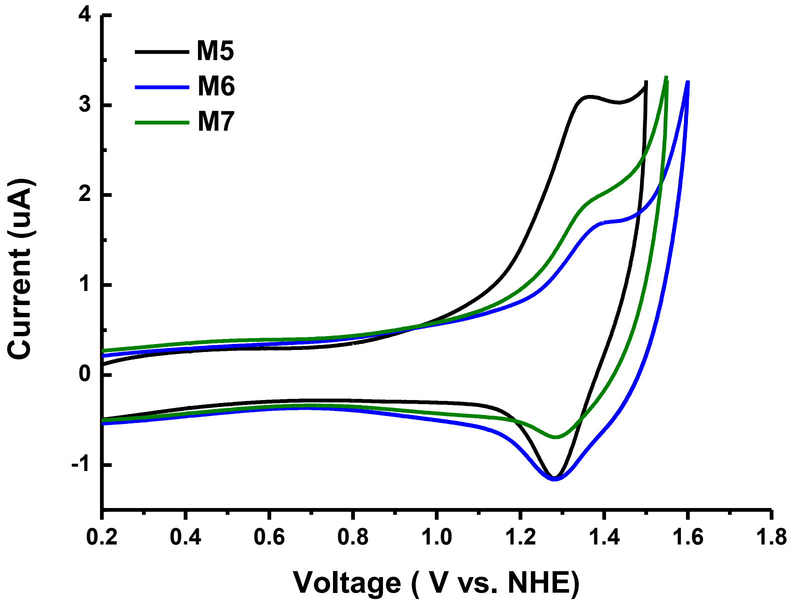

Figure S28 | CV curves of M5, M6 and M7.
